# H3K9 methyltransferases and demethylases control lung tumor-propagating cells and lung cancer progression

**DOI:** 10.1038/s41467-018-07077-1

**Published:** 2018-11-19

**Authors:** S. P. Rowbotham, F. Li, A. F. M. Dost, S. M. Louie, B. P. Marsh, P. Pessina, C. R. Anbarasu, C. F. Brainson, S. J. Tuminello, A. Lieberman, S. Ryeom, T. M. Schlaeger, B. J. Aronow, H. Watanabe, K. K. Wong, C. F. Kim

**Affiliations:** 1 Stem Cell Program, Division of Hematology/Oncology and Pulmonary and Respiratory Diseases, Children’s Hospital Boston, Boston, MA 02115 USA; 2 Department of Genetics, Harvard Medical School, Boston, MA 02115 USA; 3 Laura and Isaac Perlmutter Cancer Center, New York University Langone Medical Center, New York, NY 10016 USA; 4 Department of Toxicology and Cancer Biology, University of Kentucky, Lexington, KY 40536 USA; 5 Department of Medicine, Division of Pulmonary, Critical Care and Sleep Medicine, Tisch Cancer Institute, Icahn School of Medicine at Mount Sinai, New York, NY 10029 USA; 6 Department of Cancer Biology, Perelman School of Medicine at the University of Pennsylvania, Abramson Cancer Center, Philadelphia, PA 19104 USA; 7 Division of Biomedical Informatics, Cincinnati Children’s Research Foundation, University of Cincinnati College of Medicine, Cincinnati, OH 45229 USA; 8 Harvard Stem Cell Institute, Cambridge, MA 02138 USA

## Abstract

Epigenetic regulators are attractive anticancer targets, but the promise of therapeutic strategies inhibiting some of these factors has not been proven in vivo or taken into account tumor cell heterogeneity. Here we show that the histone methyltransferase G9a, reported to be a therapeutic target in many cancers, is a suppressor of aggressive lung tumor-propagating cells (TPCs). Inhibition of G9a drives lung adenocarcinoma cells towards the TPC phenotype by de-repressing genes which regulate the extracellular matrix. Depletion of G9a during tumorigenesis enriches tumors in TPCs and accelerates disease progression metastasis. Depleting histone demethylases represses G9a-regulated genes and TPC phenotypes. Demethylase inhibition impairs lung adenocarcinoma progression in vivo. Therefore, inhibition of G9a is dangerous in certain cancer contexts, and targeting the histone demethylases is a more suitable approach for lung cancer treatment. Understanding cellular context and specific tumor populations is critical when targeting epigenetic regulators in cancer for future therapeutic development.

## Abstract (web summary)

The effects of epigenetic regulators on different tumor cell populations can affect their potential as anticancer targets. In this study, the authors demonstrate that the histone methyltransferase G9a is a suppressor of lung tumor-propagating cells and tumor progression, acting through chromatin modification with MMP10 as one of its targets for metastasis regulation.

## Introduction

Tumors are phenotypically heterogeneous, containing cells with widely different disease-promoting potential. The most aggressive cells exhibit regenerative and proliferative behaviors associated with tissue progenitor cells and are often referred to as cancer stem cells or tumor-propagating cells (TPCs). We previously identified TPCs in the *Kras*^*LSL*^ *p53*^*flox/flox*^ (hereafter referred as *Kras;p53*) mouse model of lung adenocarcinoma. Sca-1 (stem cell antigen-1, also *Ly6a*) and CD24 enriched for a population of cells with enhanced transplantation efficiency^1^, and greater metastatic potential^2^. A combination of CD24, ITGB4 (integrin β4), and high Notch receptor expression has also been used to isolate TPCs from this model^3^. These approaches have made it possible to investigate why TPCs behave differently compared to other tumor cells.

Chromatin-modifying and/or chromatin-interacting proteins, often referred to as epigenetic regulators, play essential roles in delineating cell fate and governing the behavior of stem or progenitor cells. Epigenetic regulators are frequently amplified, mutated, or mis-regulated in cancer, often playing important roles in tumorigenesis, and have thus become the focus of an entire field of therapeutic discovery^4,5^. G9a and Glp (also *Ehmt2* and *Ehmt1*, respectively) are histone methyltransferases (HMTs) that catalyze the mono-methylation and di-methylation of histone H3 lysine 9 (H3K9). These enzymes are known for regulating embryonic stem cell differentiation, particularly into neuronal lineages^6,7^, and have also been linked to cancer, where G9a is mutated or amplified at a low frequency in a variety of tumors^8^. The majority of studies describe G9a as an oncogene, finding G9a to have pro-proliferative and pro-epithelial–mesenchymal transition (pro-EMT) functions^9–12^. These results have spurred the development of chemical inhibitors of G9a/Glp in the hope that they could be used as anticancer therapeutics^13–15^. Chromatin modifications are dynamically regulated, and multiple lysine demethylases (KDMs) can remove H3K9 methylation, including Kdm3a (also Jmjd1a, JHDM2a) and Phf8^16,17^. These enzymes have been linked to tumorigenesis and stem cell behavior in other tissues^18–20^, making them plausible candidates to regulate these phenotypes in the lung. However, a specific role for H3K9 methylation regulators in progenitor cell behavior in lung cancer has not been explored.

By screening for regulators of TPC surface markers, we identify the H3K9me1/2 HMTs G9a and Glp as TPC suppressors; depleting G9a during tumorigenesis expands the TPC population and accelerates disease progression. Inhibiting or depleting G9a upregulates genes associated with mutant KRAS and extracellular matrix (ECM) regulation, and depleting these genes or overexpressing G9a impedes tumor transplantation. Depleting or inhibiting H3K9me1/2 KDMs impairs the TPC phenotype, revealing this a possible means to further develop a lung adenocarcinoma therapeutic.

## Results

### G9a/Glp inhibition drives cells towards the TPC phenotype

**Fig. 1:**
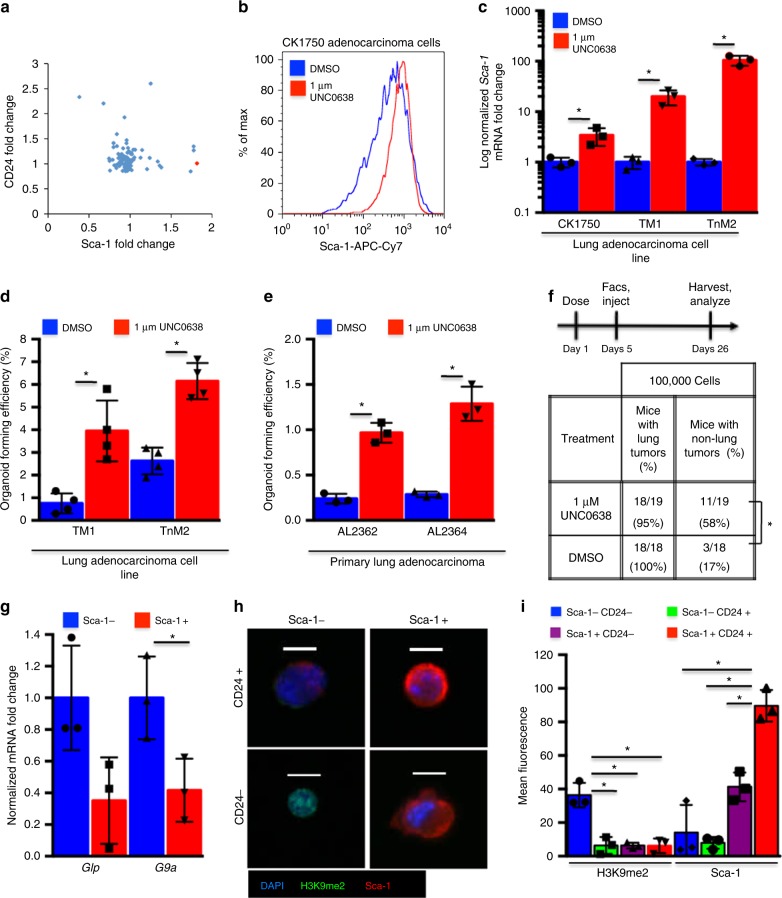
G9a/Glp inhibition drives cells towards the TPC phenotype. **a** Scatter plot of fold change in median fluorescence of drug-screened CK1750 cells labeled with fluorescent anti-CD24 and anti-Sca-1 antibodies. Each point represents an individual compound, red = UNC0638, blue = other compounds. **b** Representative (*n* = 5) FACS histogram of Sca-1 fluorescence of lung adenocarcinoma cells following 96 h. treatment with 1 µM UNC0638 or vehicle control, gated for live single cells. **c** qPCR of *Sca-1* mRNA normalized to *Gapdh* from adenocarcinoma cells following 96 h. treatment with 1 µM UNC0638 or vehicle control. Error bars denote standard deviation. *P < 0.05, *t* test, *n* = 3. **d** Bar chart of % of cells forming organoids 10 days after 1000 adenocarcinoma cells were plated in 3D culture with 1 µM UNC0638 or DMSO following 96 h. pre-treatment with UNC0638 or DMSO control. Error bars denote standard deviation. *P < 0.05, *t* test, *n* = 3. **e** Representative bar chart of % of cells forming organoids 10 days after 5000 single cells isolated from a primary adenocarcinoma were plated in 3D culture with 1 µM UNC0638 or DMSO. Error bars denote standard deviation. *P < 0.05, *t* test, *n* = 3 technical replicates. **f** Experimental design of G9ai in intravenous transplantation assay, and table of tumor incidence in recipients of UNC0638-treated or DMSO-treated adenocarcinoma cells by intravenous injection. *P < 0.05, Fisher’s exact test. **g** Bar chart of mRNA abundance of *G9a* and *Glp* normalized to *β-Actin* in Sca-1+ cells (TPCs) relative to Sca-1− cells (non-TPCs) from FACS-sorted primary adenocarcinomas, gated for single, live, CD31−, CD45− cells. Error bars denote standard deviation. *P < 0.05, *t* test, *n* = 3. **h** Representative (*n* = 3) images of isolated lung adenocarcinoma cells sorted by CD24 and Sca-1, gated for live, single, CD31−, CD45−, Epcam+ cells after cytospin and immunostained for the indicated markers. Scale bar = 20 µm. **i** Quantification of H3K9me2 and Sca-1 immunostaining of isolated lung adenocarcinoma cells sorted by CD24 and Sca-1, gated for live, single CD31−, CD45−, Epcam+ cells. Error bars denote standard deviation. *P < 0.05, *t* test, *n* = 3

To determine which molecular pathways regulate lung adenocarcinoma TPCs, we tested a library of stem cell-regulating small molecules (Table 1). We screened a TPC-like cell line, CK1750^2^, for changes in the surface antigens Sca-1 and CD24, reasoning that changes in these markers could correspond to changes in TPC activity. Multiple compounds increased the levels of at least one TPC antigen (Fig. 1a, Supplementary Fig. 1a), and we focused on characterizing the effect of the molecule that increased Sca-1 the most significantly (1.8-fold vs. dimethyl sulfoxide (DMSO), *P* = 0.0015, *t* test), UNC0638. UNC0638, a potent specific inhibitor of the H3K9 mono-methyltransferase and di-methyltransferases Glp and G9a^21^, increased Sca-1 in multiple adenocarcinoma cell lines, with a greater difference in lines with low endogenous Sca-1 levels, TM1 and TnM2 (Fig. 1b, Supplementary Fig. 1b). UNC0638 also increased *Sca-1* mRNA (2.1-fold, *P* = 0.036, 19.8-fold, *P* = 0.0005, and 104.3-fold, *P* = 0.0001, *t* test), implying that higher Sca-1 levels were due to upregulated transcription (Fig. 1c).

**Table 1:** Composition of the Stem Cell Chemical Library used for screening lung adenocarcinoma TPCs

Compound	Mechanism	Compound	Mechanism
CCPA	A1+	Compound W	Notch
SQ22536	Acase−	DAPT	Notch
Forskolin	Acase+	DLPC	nr2a5
SR1	ahr−	2-Phospho-l-ascorbic acid	P
ITE	AHR+	IPA-3	Pak−
ABT 702 dihydrochloride	AK−	EB-47	parp1−
KP372-1	AKT−	DMPQ dihydrochloride	pdgf r inh
(+)-JQ1- 1 mg	BET−	SU6656	pdgf/fgf/vegf r inh
Dorsomorphin	bmpr−	Mycophenolate mofetil	PDH2
CITCO	CAR	PS48	PDK1
Indomethacin	cox	16,16-Dimethyl prostaglandin E2	PGE2
AMD3100	cxcr4−	GDC-0941	pi3k
FCCP	decoupl	PI-103	pi3k− mtor− dnapk−
A77 1726	dhodh	cAMPS-Rp	PKA−
RG 108	DNMT	GSK 0660	PPARd−
5-Aza-2′-deoxycytidine	DNMT−	BADGE	pparg−
5-Azacytidine	DNMT1−	GW9662	PPARg−
SGC 0946	DOT1L−	Troglitazone	pparg+
AG 1478	EGFR	AM 114	Proteasome
Erlotinib	egfr−	NSC 23766	RAC
GSK126	EZH2−	BMS 493	RAR+
PF 573228	FAK	BMS195614	RARa−
PD161570	fgfr−	AM580	RARa+
UNC 0638	g9a inh	AC 261066	RARb+
Dexamethasone	gc	CD2665	RARbg−
Depudecin	HDAC−	BMS 961	RARg+
Sodium butyrate	HDAC−	y27632	rock−
Trichostatin A	HDAC−	Blebbistatin	rock/mlck
Valproic acid	HDAC−	BI-D1870	RSK
GANT 61	HH−	W146	s1p1−
Purmorphamine	HH+	SEW2871	s1p1+
20(*S*)-Hydroxycholesterol	HH+	Splitomycin	sirp2−
IOX2	HIF+	ex527	sirt1−
IKK16	IKK	agk2	sirt2−
Revstarol	jak 3/2/1 inh	A83-01	tgfb−
cp690550	jak−	repsox	tgfb− alk5−
Tranylcypromine HCl	LSD1−	IDE-1	TGFb+
PD98059	MAPK	SB431542	tgfb1
Nutlin	mdm2 inh	TLR3/dsRNA complex inhibitor	tlr3 inh
PD0325901	MEK	MLS000389544	TR−
Rapamycin	mtor	1–850	TR+
Honokiol	nfkb−	3,3′,5-Triiodo-l-thyronine sodium salt	TR+
Betulinic acid	nfkb+	RN 1734	TRP channels
SNAP	NO	ZM 306416 hydrochloride	vegf r inh
DETA NONOate	NO+	IWP-2	wnt−
l-NAME	NOS−	XAV 939	Wnt−
		CHIR99021	wnt+gsk−

We have previously demonstrated that CD24 and Sca-1 mark a population enriched in TPCs^1,2^. To further explore the effects of G9a/Glp inhibition (hereafter referred as G9ai) on tumor-propagating ability, we modeled this phenotype with primary adenocarcinoma cells in Matrigel air–liquid interface cultures. Sca-1+CD24+ cells formed organoids at a higher rate than other primary tumor populations (5.8 vs. 3.1, 3.3, and 1.9%, *P* = 0.002, *P* = 0.0007, *P* = 0.0002, *t* test), demonstrating that more efficient in vitro organoid formation correlates with a TPC-enriched population (Supplementary Fig. 1c). G9ai of Sca-1-low adenocarcinoma cell lines increased the proportion of Sca-1-expressing cells and led to increased organoid-forming efficiency (3.95 vs. 0.75%, *P* = 0.004 and 6.1 vs. 2.7%, *P* = 0.025, *t* test) (Fig. 1d). G9ai of unsorted primary adenocarcinoma cells in 3D culture also increased organoid formation (0.97 vs. 0.25%, *P* = 0.027, 1.32 vs. 0.29%, *P* = 0.03, *t* test) (Fig. 1e) and resulted in more Sca-1+ cells when cultures were analyzed at the experimental endpoint (Supplementary Fig. 1d). To further demonstrate that G9ai could promote a TPC phenotype, we inhibited Sca-1-low adenocarcinoma cell lines and intravenously injected them into immunocompromised (nude) recipient mice (Fig. 1f, Supplementary Fig. 2a). At the experimental endpoint, we detected lung tumors in the recipients of both G9ai and vehicle control-treated cells (Fig. 1f, Supplementary Fig. 2b). However, mice that had received G9ai cells more frequently presented with tumors outside the lung (thoracic lymph nodes, aorta, subcutaneous) (58 vs. 17%, *P* = 0.017, Fisher’s exact test) (Fig. 1f, Supplementary Fig. 2b), demonstrating that G9ai can also promote a more tumorigenic TPC phenotype in vivo.

Our findings that lower G9a and H3K9me2 were associated with TPCs and that G9ai can increase the tumorigenicity of cancer cells appeared to contrast with other studies, suggesting that G9a is oncogenic^9–12^. Although 1 µM UNC0638 (the concentration used in our assays) did not affect cell proliferation or survival (Supplementary Fig. 2c, d), higher concentrations (5 and 10 µM) resulted in significant decreases (optical density (OD) 600 1.16 vs. 0.74, 0.09, *P* = 0.002, <0.001, *t* test, Supplementary Fig. 2d). This was in line with previous findings describing G9 as a pro-proliferative^10,12^, and shows how without considering cellular context and tumor heterogeneity, G9ai could be considered as a potential anti-oncogenic treatment.

As enzymatic inhibition of G9a/Glp could promote TPC characteristics in adenocarcinoma cells, we hypothesized that less G9a/Glp or deregulated H3K9me1/2 could be an intrinsic TPC property. Re-analysis of our previous gene expression data comparing *Kras;p53* TPCs vs. non-TPCs^2^ indicated that *G9a*, but not *Glp*, was significantly downregulated in the TPC-enriched population (0.41-fold, *P* = 0.037, *t* test) (Fig. 1g), suggesting that reduced G9a levels may be important to lung TPCs. To confirm this association, we stained global H3K9me2 and Sca-1 in sorted lung adenocarcinoma populations. We found that global H3K9me2 was significantly higher in the least tumorigenic, Sca-1−CD24− cell population than in the CD24+Sca-1-, CD24-Sca-1+ and CD24+Sca-1+ populations (36.3 fluorescent units vs. 6.4, 4.8, 6.2, *P* = 0.004, *P* = 0.003, and *P* = 0.004, *t* test), while Sca-1 was significantly higher in the Sca-1+CD24+ population compared to all the others (89.5 vs. 14.0, 9.6, 33.4, *P* = 0.003, *P* = 0.003, and *P* = 0.013, *t* test) (Fig. 1h, i). These data show an inverse association between Sca-1, TPC, and H3K9me2, suggesting that H3K9 demethylation may be a feature of, or a prerequisite for, lung adenocarcinoma TPCs and tumor progression and metastasis.

### G9a depletion promotes tumor progression and metastasis

**Fig. 2:**
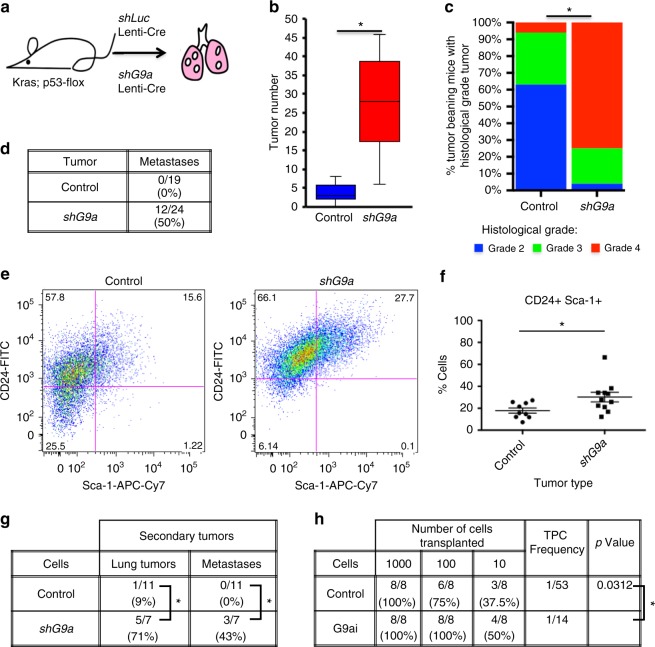
G9a depletion expands the TPC population and promotes tumor progression and metastasis. **a** Cartoon of experimental design to deplete G9a in Cre-induced adenocarcinomas. **b** Box and Whisker plot of tumor number per mouse, in recipients of control and *shG9a* lentiviruses. Boxes represent inner quartiles, center line = median, whiskers = 1.5 × IQR. *P < 0.05, Mann–Whitney test. **c** Bar chart of tumor grade/mouse of control and *shG9a* recipients. *P < 0.05, *χ*^2^ test, *n* = 16 and *n* = 24 mice, respectively. **d** Table of metastasis occurrence in the recipients of control and *shG9a* lentiviruses. *P < 0.05, Fisher’s exact test. **e** Representative FACS plots of dissociated tumors from control (*n* = 9) and shG9a (*n* = 11) recipients, gated for live, single, CD31−, CD45−, Epcam+ cells. **f** Chart showing the median proportion of CD24+Sca-1+ TPCs in control and shG9a tumors determined by FACS. Each data point represents tumors from an individual mouse. *P < 0.05, Mann–Whitney test. **g** Table of secondary tumor and metastasis incidence and location in recipients of orthotopically transplanted control and *shG9a* tumor cells. *P < 0.05, Fisher’s exact test. **h** Table of tumor incidence and TPC frequency in recipients of control or G9ai adenocarcinoma cells in limiting dilution assay. TPC frequency and *P* value (*χ*^2^) were calculated by ELDA software

Historically, TPCs have largely been studied ex vivo using surface markers and transplantation experiments. Identification of G9a as a TPC-regulating enzyme allowed us to test its function, and the role of TPCs more broadly, in tumorigenesis in situ. We utilized bi-functional Cre-U6shRNA lentiviral vectors to knockdown G9a only in the induced tumors of *Kras;p53* recipient mice (Fig. 2a, Supplementary Fig. 3a). At the experimental endpoint, recipients of *shG9a* lentivirus had remarkably progressed disease compared to *shLuciferase* controls, with significantly increased tumor number (*P* = 0.0005, *t* test) and tumor burden (Fig. 2b, Supplementary Fig. 3b). Histological examination of tumors revealed that *shG9a* recipients had significantly advanced disease (*P* < 0.0001, *χ*^2^ test), with the majority of mice presenting aggressive, poorly differentiated, grade 4 tumors, while the majority of control mice had only grade 2 tumors (Fig. 2c, Supplementary Fig. 3c). Quantitative PCR (qPCR) of isolated tumor cells and immunostains of lung tumor sections from these mice confirmed that G9a was significantly depleted in *shG9a* recipient tumors compared to controls (Supplementary Fig. 3d, e). Although we sacrificed mice in the same time frame for comparison, *shG9a* mice grew sicker more quickly than controls (*P* = 0.017, Mantel–Cox log-rank test), suggesting that G9a depletion was not simply selecting for escaped, more aggressive tumor cells (Supplementary Fig. 3f). Proliferation was not altered in endpoint *shG9a* tumors; similar proportions of cells stained for Ki67 in *shG9a* and control tumors (21.1 vs. 14.6%, *P* = 0.45, *t* test) (Supplementary Fig. 4a, b).

Further analysis of *shG9a Kras;p53* mice supported our prediction that *shG9a* would correlate with more metastasis, as we previously found that CD24+Sca-1+ cells give rise to significantly more metastases after orthotopic transplantation compared to other tumor cell populations^2^. Indeed, metastases were observed in the thoracic pleura and lymph nodes in a significantly higher proportion of *shG9a Kras;p53* primary mice compared to controls (50 vs. 0%, *P* = 0.0003 and 25 vs. 0%, *P* = 0.0265, Fisher’s exact test, Fig. 2d). Furthermore, fluorescence-activated cell sorting (FACS) of metastases from *shG9a Kras;p53* mice or *Kras;p53* mice with tumors initiated with Adeno-Cre revealed that they almost entirely consisted of CD24+Sca-1+ TPCs (Supplementary Fig. 4c–e). Together, the data suggest that G9a depletion can drive lung adenocarcinoma progression and promote metastasis.

### G9a depletion expands the TPC population in vivo

FACS analysis of dissociated tumors revealed differences in the sub-populations of *shG9a* and control tumors. We found a 1.8-fold significant increase in the CD24+Sca-1+ cell population, previously shown to be enriched in TPCs^2^, in *shG9a* tumors (27.7 vs. 15.6%, *P* = 0.0185, Mann–Whitney test) and a corresponding decrease in the proportion of CD24−Sca-1− cells (28 vs. 18.2%) (Fig. 2e, f), reinforcing our hypothesis of G9a as a negative regulator of lung TPCs. It has also been previously reported that Notch3 is enriched in lung TPCs and is required for lung TPC function^3^. In line with these findings, we observed a significant increase in tumors with nuclear Notch3 localization in *shG9a* vs. *shLuc* recipients and in mice receiving high titer Cre-Adenovirus, which display more grade 3 and grade 4 tumors than *shLuc* lentivirus controls (49 vs. 10%, *P* = 0.001, 26%, *P* = 0.008, Mann–Whitney test) (Supplementary Fig. 4f, g).

In orthotopic transplantation experiments, recipients of *shG9a* cells more frequently developed lung tumors and metastases (71 vs. 9%, *P* = 0.0095, 43 vs. 0%, *P* = 0.043, Fisher’s exact test) compared to mice that received control *Kras;p53* cells, further confirming that *shG9a* tumors have enriched TPC (Fig. 2g). To show that G9a could directly increase the frequency of TPCs, we used G9ai on wild-type lung adenocarcinoma cells and performed limiting dilution transplantation assays. G9ai cells initiated more tumors at higher dilutions than controls, increasing the estimated TPC frequency (1/14 vs. 1/53, *P* = 0.0312, *χ*^2^ test) (Fig. 2h). These results suggest that G9a loss drives lung adenocarcinoma progression and metastasis by increasing the proportion of TPCs within the tumor.

### G9a controls TPCs through KRAS-associated and ECM-associated genes

To understand how G9 controls TPC phenotypes, we sought to identify genes that acutely respond to changes in G9a by using G9ai and H3K9me2 chromatin immunoprecipitation-sequencing (ChIP-seq) and RNA-sequencing (RNA-seq). As has been observed with other cells, we found that H3K9me covered large, Mb-sized genomic domains^22,23^. We did not observe changes in the locations of these domains (Supplementary Fig. 5a), even though global H3K9me2 levels were significantly reduced (Supplementary Fig. 5b), suggesting that G9ai reduced H3K9me2 evenly across the genome. RNA-seq however identified many genes differentially expressed by G9ai (Supplementary Fig. 5c). ChIP-seq showed lower H3K9me2 across the transcription starting sites (TSS) of the RNA-seq genes commonly upregulated across multiple cell lines, and higher H3K9me2 at the commonly downregulated genes (Supplementary Fig. 5d).

**Fig. 3:**
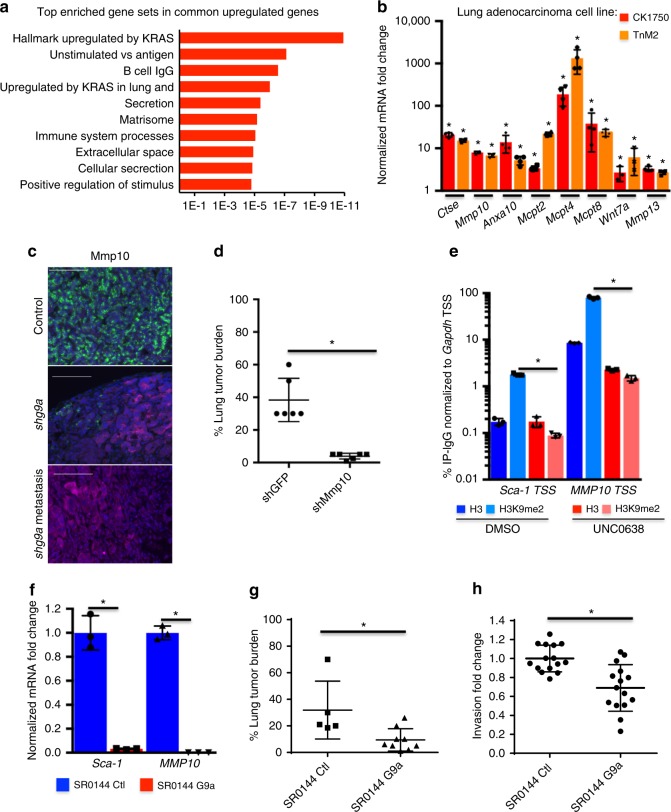
G9a controls TPCs through KRAS-associated and ECM-associated genes. **a** Molecular Signatures Database enrichment of gene terms associated with genes upregulated upon G9a/Glp inhibition. **b** Bar chart of fold change of *Gapdh*-normalized mRNA levels of indicated genes in adenocarcinoma cells treated with 1 µM UNC0638 relative to DMSO-treated controls. Error bars denote standard deviation. *P < 0.05, Fisher’s *t* test, *n* = 4. **c** Representative (*n* = 5) images of *shLuc* control, and *shG9a* lung tumors and *shG9a* metastases immunostained for the indicated proteins. Scale bar = 100 µm. **d** Chart of % lung tumor area per H&E-stained section from recipients of intravenously injected *shGFP*, and *shMmp10* SR0144 *shG9a* lung adenocarcinoma cells. *P < 0.05, Fisher’s *t* test. **e** Representative bar chart of % immunoprecipitation compared to input DNA–IgG of H3 and H3K9me2 ChIPs at the indicated TSS relative to *Gapdh* TSS of lung adenocarcinoma cells treated with 1 µM UNC0638 or DMSO. Error bars denote standard deviation. *P < 0.05, Fisher’s *t* test, *n* = 3 technical replicates. **f** Bar chart of mRNA from the indicated genes normalized to *Gapdh* from control and G9a-overexpressing SR0144 *shG9a* lung adenocarcinoma cells. Error bars denote standard deviation, *P < 0.05, Fisher’s *t* test, *n* = 3. **g** Chart of % lung tumor area per H&E-stained section from recipients of intravenously transplanted control and G9a-overexpressing SR0144 *shG9a* lung adenocarcinoma cells. *P < 0.05, Fisher’s *t* test. **h** Chart of fold change in invasion through a 3D matrix-coated migration transwell of G9a-overexpressing relative to control SR0144 *shG9a* lung adenocarcinoma cells. *P < 0.05, Fisher’s *t* test

Gene ontology enrichment analysis revealed that the commonly upregulated genes were most significantly enriched in signatures associated with expression of oncogenic KRAS, as well as genes in signatures associated with the ECM, cellular secretion, and the extracellular space (Fig. 3a, Supplementary Fig. 5e). Additionally, the genes enriched within these terms were amongst the individual most upregulated genes, and several genes were present in both the KRAS-associated and ECM terms (Supplementary Fig. 5e). We narrowed our focus to the most significantly upregulated genes enriched in the KRAS-associated and ECM terms as candidates to mediate the TPC phenotype. We confirmed the significant upregulation of multiple genes upon G9ai by qPCR across multiple adenocarcinoma cell lines (Fig. 3b, Supplementary Fig. 5f). To confirm that these genes were also regulated by G9a in vivo, we stained sections of control and *shG9a* tumors. Matrix metalloproteases 10 and 13 (Mmp10 and Mmp13) were expressed in *shG9a* metastases and in *shG9a* primary tumors, but were absent in control tumors (Fig. 3c, Supplementary Fig. 6a). We also confirmed by qPCR that a subset of these genes (*Anxa10*, *Ctse*, *Mmp10*, *Mmp13*, *Lcp1*) were consistently upregulated in *shG9a* tumor cells compared to controls (Supplementary Fig. 6b).

Of these genes, Mmp10 was the highest ranked gene enriched in both the KRAS-associated and ECM gene signatures. Interestingly, Mmp10 has previously been reported as required for lung stem cell expansion as well as lung adenocarcinoma initiation and metastasis^24,25^, making it a logical candidate to mediate the TPC phenotypes regulated by G9a. We depleted Mmp10 from cell lines derived from *shG9a* tumors with shRNA hairpins (Supplementary Fig. 6c) and compared their tumor-forming efficiency with *shGFP* controls. Depletion of Mmp10 in the *shG9a* background significantly reduced tumor burden following intravenous transplantation (38.3 vs. 3.9%, *P* < 0.0001, *t* test) (Fig. 3d), implying that Mmp10 functions in the TPC phenotype. Furthermore, ChIP-PCR showed that H3K9me2 was significantly depleted by G9ai at the TSSs of *Mmp10* as well as *Sca-1* (*P* = 0.004, *P* = 000.5, *t* test) (Fig. 3e), suggesting that transcription of these genes was directly regulated by G9a.

To confirm that G9a controlled the TPC phenotype by suppressing KRAS-associated and ECM-regulating genes, we rescued *G9a* expression in SR0144 cells derived from *shG9a* tumors. Expression of human G9a increased global H3K9me2 compared to Cre-expressing controls (Supplementary Fig. 6d, e). G9a rescue significantly repressed G9a-regulated genes *Sca-1* and *Mmp10* (0.03-fold, *P* = 0.0003 and 0.0008-fold, *P* < 0.0001, *t* test) (Fig. 3f), lowered Sca-1 surface protein levels (Supplementary Fig. 6f), and significantly reduced Mmp enzymatic activity (0.69-fold, *P* = 0.0082, *t* test) (Supplementary Fig. 6g). Following intravenous transplantation, G9a-rescued cells generated a significantly lower lung tumor burden than controls (9.4 vs. 31.9%, *P* = 0.016, *t* test) (Fig. 3g), reinforcing the conclusion that G9a directly regulates TPC tumorigenicity. As G9a represses Mmp10 and other secreted proteases, we asked if G9a could regulate how tumor cells interact with the ECM. When we cultured cells in invasion assays, where serum-starved cells must migrate through a 3D matrix barrier towards serum-rich media, G9a-depleted cells showed significantly more invasion than controls expressing G9a (0.69-fold, *P* = 0.0002, *t* test) (Fig. 3h). There was no difference between cells in a matrix-free migration assay (Supplementary Fig. 6h), suggesting that G9a activity more specifically regulates genes that impact the ability of cells to manipulate the matrix. In total, these data support the conclusion that one way by which G9a suppresses TPC activity is by repression of Mmp10, which in turn influences the ability to degrade the ECM.

### Antagonizing H3K9 KDMs represses TPC phenotypes

**Fig. 4:**
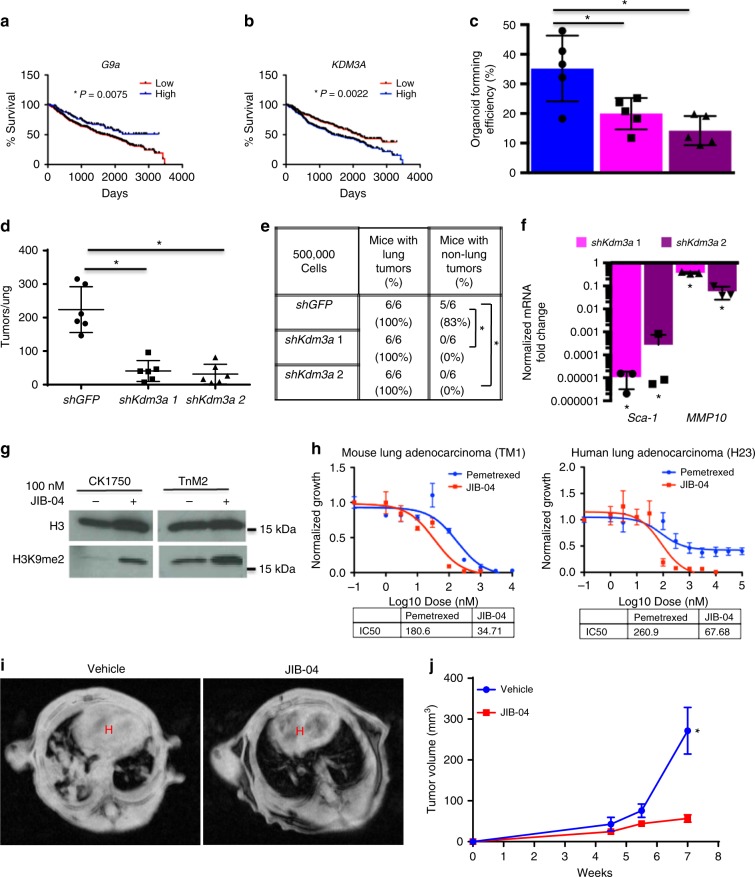
Antagonizing H3K9 KDMs represses TPC phenotypes. **a**, **b** Kaplan–Meier curves of 10-year survival of patients from the director’s challenge cohort of early stage lung adenocarcinoma. Populations sorted for high and low expression of **a** H3K9me1/2 G9a and **b** H3K9me1/2 demethylase KDM3A. *P < 0.05, Mantel–Cox log-rank test. **c** Bar chart of % of cells forming organoids 10 days after 1000 *shGFP* and *shKdm3a* CK1750 adenocarcinoma cells were plated in 3D culture. Error bars denote standard deviation. *P < 0.05, Fisher’s *t* test, *n* = 4. **d** Chart of average number of tumors per H&E-stained section from recipients of intravenously transplanted *shGFP* and *shKdm3a* adenocarcinoma cells. *P < 0.05, Mann–Whitney test. **e** Table of transplanted mice with lung and non-lung tumors. *P < 0.05, Fisher’s exact test. **f** Bar chart of fold change of *Gapdh*-normalized mRNA levels of indicated genes in *shKdm3a* adenocarcinoma cells over *shGFP* control cells. Error bars denote standard deviation. *P < 0.05, Fisher’s *t* test, *n* = 3. **g** Western blot of whole cell lysates from CK1750 adenocarcinoma cells treated with either 100 nM JIB-04 or DMSO for 96 h, and then immunoblotted for the indicated antibodies. **h** Representative dose–response curve of mouse (TM1) and human (H23) NSCLC cells grown for 96 h with JIB-04 or Pemetrexed, normalized to growth with vehicle control. **i** Representative MR images of allograft mice treated with vehicle or JIB-04 at 7 weeks post transplantation. H indicates heart area. **j** Chart of average tumor volume of allografted mice treated with either vehicle or JIB-04. *P < 0.05, Fisher’s *t* test

*G9a/Glp* are not commonly mutated in lung adenocarcinoma^26^; therefore, we analyzed *G9a* and *Glp* expression in the Directors Challenge data set from over 400 adenocarcinoma patients to determine if G9a may be linked to clinical criteria in patients. *Glp* expression was not predictive of outcome (Supplementary Fig. 7a), yet higher *G9a* expression was correlated with significantly better 10-year survival (*P* = 0.0075, Mantel–Cox log-rank test) (Fig. 4a), as we would predict if G9a functioned as we have shown in the mouse. Interestingly, this contrasts with some previous studies, which have suggested that *G9a* overexpression is associated with worse clinical outcome^9,27^. As chromatin modifications are bi-directionally regulated, we looked to see if any H3K9me1/2 KDMs were also predictive of clinical outcome. We found one H3K9 KDM, *KDM3A* (also *JMJD1A*, *JHDM2A*) for which high expression correlated with worse 10-year survival (*P* = 0.0022, Mantel–Cox log-rank test) (Fig. 4b), consistent with our hypothesis that lower H3K9me1/2 promotes a TPC phenotype and accelerated disease. To test if antagonizing an H3K9me1/2 KDM might impair TPC phenotypes and improve outcome, we depleted *Kdm3a* in CK1750 mouse adenocarcinoma cells (Supplementary Fig. 7b). We observed that *shKdm3a* cells displayed lower organoid-forming efficiency than control *shGFP* cells in 3D culture (Fig. 4c), despite their proliferation rate in adherent culture being comparable to controls (Supplementary Fig. 7c). When *shKdm3a* cells were transplanted into mice, significantly fewer lung and extra-lung tumors formed compared to control cells (Fig. 4d, e), suggesting that the TPC properties of these cells were also impaired. Similarly to rescued *shG9a* adenocarcinoma cells (Fig. 3f), we observed significant downregulation of the G9a targets *Sca-1* and *Mmp10* (Fig. 4f), suggesting that Kdm3a depletion may impede TPCs through the same mechanisms as *G9a* expression in lung adenocarcinoma cells. When we returned to our primary mouse adenocarcinoma data, we found that a different H3K9me1/2 demethylase, *Phf8*, was significantly upregulated in Sca-1+ TPCs (3.6-fold, *P* = 0.022, *t* test) (Supplementary Fig. 7d). We also depleted *Phf8* in CK1750 cells (Supplementary Fig. 7e, f), and found that similarly *shPhf8* cells had a lower organoid-forming efficiency (Supplementary Fig. 7g), formed fewer tumors when transplanted (Supplementary Fig. 7h), and had reduced *Sca-1* and *Mmp10* expression (Supplementary Fig. 7i).

Currently, there are no inhibitors that specifically target Kdm3a or Phf8; however, there are several broad-spectrum KDM inhibitors, which will inhibit these enzymes along with other KDMs. We found that pan-jumonji inhibitor JIB-04^28^ increased H3K9me2 levels (Fig. 4g), and also resulted in the loss of surface Sca-1 in adenocarcinoma cells (Supplementary Fig. 7j). Furthermore, JIB-04 affected the viability of both mouse and human adenocarcinoma and squamous non-small-cell lung cancer (NSCLC) cell lines. JIB-04 had an approximately equal or lower half-maximal inhibitory concentration (IC_50_) in both mouse and human cell lines than Pemetrexed, a current frontline therapeutic for the treatment of NSCLC (34.71 vs 180.6 nM, 67.68 vs 260.9 nM, Fig. 4h). We also performed allograft transplantations of adenocarcinoma cells into immunologically competent host mice and treated them with either vehicle or JIB-04. After 7 weeks, relative tumor volume was significantly higher in the vehicle cohort compared to JIB-04-treated mice (271.35 vs. 56.7 mm^3^, *P* = 0.0014, *t* test) (Fig. 4i, j), suggesting that targeting TPCs through H3K9me KDM inhibition could be an effective therapy for lung adenocarcinoma.

## Discussion

**Fig. 5:**
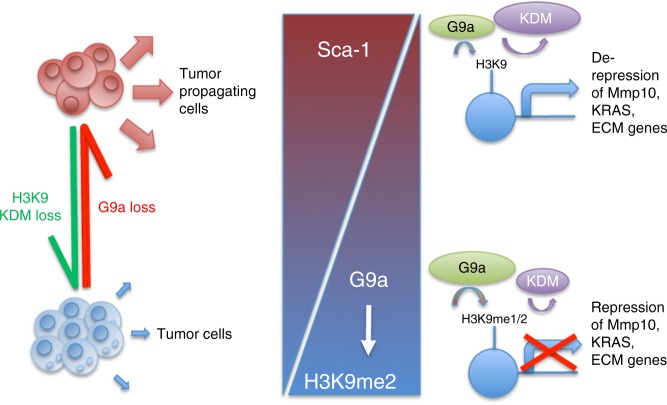
H3K9 methyltransferases and demethylases control lung tumor-propagating cells through transcriptional regulation of Mmp10, KRAS and ECM genes. In lung adenocarcinoma non-stem tumor cells, G9a is relatively highly expressed and H3K9me2 KDMs are relatively under-expressed. Chromatin is relatively enriched in H3K9me2, which represses the transcription of Sca-1, Mmp10, and other genes associated with mutant KRAS and ECM regulation. Loss of G9a or gain of H3K9me2 KDMs reduces H3K9me2 and allows for the expression of previously repressed genes. This enables tumor cells to acquire the tumor-propagating cell phenotype, and the expansion of the tumor-propagating cell population drives lung adenocarcinoma progression and metastasis. Re-expression of G9a or antagonism of H3K9me2 KDMs can reverse the TPC phenotypes, mitigating lung adenocarcinoma progression

We found that inhibition of G9a, an HMT previously reported to have oncogenic activity, leads to enhanced TPC function and drives metastatic tumor progression. In vitro and in vivo studies revealed that G9a represses a gene signature highly associated with mutant KRAS-regulating and ECM-regulating genes, including Mmp10, which partially mediates the tumorigenicity of G9a-depleted tumor cells. Conversely, we found that inhibition of H3K9 KDMs (KDMi) may be a beneficial treatment for advanced lung adenocarcinoma. By studying the epigenetic dependencies of TPCs that represent a minority of tumor cells, our work has revealed a potential new therapeutic intervention for advanced lung cancer (Fig. 5).

Since the discovery of potent G9a/Glp inhibitors, there has been an increase in studies investigating these enzymes in cancer. Indeed, much of the rationale for developing G9a/Glp inhibitors was that many tumors stained positively for these enzymes and this was often correlated with worse outcome^14,15,29^. It has been observed amongst almost all cancer models tested that G9a inhibition has antiproliferative effects, and different mechanisms have been proposed^9,10^. While we found that high concentrations of G9a inhibitors were antiproliferative, we also observed that G9a is downregulated in highly tumorigenic TPCs (and in an H3K9 KDM, Phf8 is upregulated), coinciding with a significant reduction in global H3K9me2. Our data suggest that downregulation of G9a promotes TPCs, leading to disease progression, metastasis, and worse clinical outcome, thus raising caution for the use of G9a inhibitors, particularly for lung cancer treatment.

Our studies suggest that TPCs can promote tumorigenesis and metastasis by influencing the extracellular environment. While our data are consistent with previous studies which found that deleting or inhibiting G9a can increase stem cell or tumor stem-like populations and oppose tissue-specific differentiation^23,30^, we focused this study on the role of G9a in regulation of genes affecting ECM. Mmp10, the G9a target we characterized in lung cancer cells, directly mediates TPC tumorigenicity. In addition to degrading the ECM to facilitate invasion, Mmp10 has also been proposed to promote metastasis by proteolytically regulating extracellular pools of growth factors such as TGF-β and Wnt^24,25,31,32^. While at this stage we cannot rule out an Mmp10 effect on these signaling pathways, we favor the hypothesis that Mmp10 expressed by lung TPCs acts on the ECM, as we observed that G9a-overexpressing cells repress Mmp10, have lower Mmp enzymatic activity, and are partially impaired in invading through a 3D matrix. It has been hypothesized that the tumor microenvironment influences TPCs, but the ability of TPCs to alter their surroundings via ECM remodeling warrants further study.

Mmps are strongly associated with poor survival in lung cancer, but to date trials of Mmp inhibitors have not proven successful, through the lack of efficacy, toxicity, or poor bioavailability^33^. We found that Mmp10 is de-repressed by H3K9 KDMs; therefore, KDMi may prevent or delay the re-expression of these genes, potentially providing another angle to target Mmps in lung cancer. Other studies have also found that KDMs are upregulated in cancers and this correlates with worse outcome^28,34^. This further suggests that KDMi could be a useful therapeutic intervention, particularly as chemoresistance is a proposed TPC trait^35^. JIB-04 is a nonspecific KDM inhibitor and it is unlikely that all the effects we observe are due to its inhibition of H3K9 KDMs. While several more specific KDM inhibitors exist^36,37^, there are none that inhibit all the H3K9me KDMs. The development of pan H3K9me KDM inhibitors would be of great interest and potentially therapeutic benefit for highly refractive diseases such as lung adenocarcinoma. As *KRAS* mutations are common, but KRAS itself is not a druggable target, our finding that H3K9 KDMi decreases expression of genes associated with oncogenic KRAS suggests that H3K9 KDMi represents a viable personalized therapy for patients with *KRAS*-mutated adenocarcinoma.

This study has highlighted the importance of choosing the best model of cellular heterogeneity for analyses that may lead to new therapeutic approaches in cancer. Studies primarily based on cell lines or whole tumors have suggested that G9a is oncogenic and a good target for epigenetic therapy, but our approach, beginning with analyzing different tumor populations, led to a startlingly different conclusion. We propose that analyzing the effects of new therapeutics on different tumor populations, especially the most tumorigenic stem-like cells, would be greatly beneficial. This may increase the success rate of clinical trials and thus reduce the cost and time taken to bring new cancer therapeutics to the patient.

## Methods

### Cell lines

Mouse cell lines CK1750, SC241, and SR0144 were generated as previously described^2^. Cell lines TM1 and TnM2 were previously provided by Monte Winslow. Mouse cell lines were cultured in Dulbecco's modified Eagle's medium (DMEM) + 10% fetal bovine serum (FBS), 4mM l-glutamine, and penicillin/streptomycin at 37 °C with 5% CO_2_. Human H23 cells (ATCC CRL-5800) were used as previously described^38^, cultured in RPMI-1640 media with 10% FBS, 4mM l-glutamine, and 50 U/ml penicillin/streptomycin at 37 °C with 5% CO_2_.

### Vectors

The pLKO.1 *Mmp10*, *Kdm3a* and *Phf8* shRNA construct clones TRCN000031240, TRCN000031241, TRCN0000252743, TRCN0000252745, TRCN0000086826and TRCN0000086827 were purchased from Sigma and the *shGFP* plasmid 12273 was available from Addgene^38^. The *G9a* expression vector^39^ 41721 and Cre expression control vector^40^ 34565 were also purchased from Addgene. The shRNA EHMT2 (G9A) lentiviral vectors were created by cloning hairpin oligonucleotides into the U6shRNA pgkCre backbone, created by the Tyler Jacks lab and available from Addgene as previously described^41^. The vectors used were as follows: *shLuciferase*—FWD, 5′-5Phos/TGA GCT GTT TCT GAG GAG CCT TCA AGA GAG GCT CCT CAG AAA CAG CTC TTT TTT C-3′ and REV, 5′-5Phos/CGA GAA AAA AGA GCT GTT TCT GAG GAG CCT CTC TTG AAG GCT CCT CAG AAA CAG CTC A-3′; *shEHMT2 1*—FWD, 5′-5Phos/TGG CTG CTC CAG GAG TTT AAT TCA AGA GAT TAA ACT CCT GGA GCA GCC TTT TTT C-3′ and REV, 5′-5Phos/TCG AGA AAA AAG GCT GCT CCA GGA GTT TAA TCT CTT GAA TTA AAC TCC TGG AGC AGC CA-3′; *shEHMT2 2*—FWD, 5′-5Phos/TGG AAG AGG AGG AAG AAG AAT TCA AGA GAT TCT TCT TCC TCC TCT TCC TTT TTT C-3′ and REV, 5′-5Phos/TCG AGA AAA AAG GAA GAG GAG GAA GAA GAA TCT CTT GAA TTC TTC TTC CTC CTC TTC CA-3′.

Generation, concentration, and infection of mice with lentivirus was performed as previously described^42^.

### Chemical compounds

The Stem Cell Chemical Library was assembled from commercially available compounds, and diluted to a stock of 10 mM in DMSO. Chemicals were further diluted to 20 µM in DMEM before being added to the cells. The chemicals used are listed in Table 1. UNC0638 was purchased from Cayman Chemical and diluted to a stock of 10 mM in DMSO for all cell culture experiments. JIB-04 was purchased from Tocris was diluted to a stock of 10 mM in DMSO for cell culture experiments and 5.5 mg/ml in Koliphor-EL (Sigma) for mouse experiments. Pemetrexed was purchased from the Dana Faber Cancer Center and was diluted to a stock of 100 mM in ddH_2_O. For cell culture experiments, cells were grown in the presence of the drug for 96 h with fresh media and drugs were supplied at 48 h.

### Flow cytometry of in vitro cultured cells

For flow cytometry screening, CK1750 cells were plated at 300 cells/well in 96-well plates and treated with compounds from the Chemical Library for 96 h. For routine flow cytometry analysis, cells were grown and cultured as described above. Cells were then dissociated, washed, and re-suspended in 2% FBS in phosphate-buffered saline (PBS) (PF2) and stained with anti-CD24-PE (BD Pharmingen 553262, 1:100), anti-Sca-1-APC-Cy7 (BD Pharmingen 560654, 1:100), and DAPI (4′,6-diamidino-2-phenylindole) for 15 min on ice. Cells were washed again and re-suspended in 50 mM EDTA, 2% FBS in PBS, filtered (40 µM), and sorted on a BD LSR II flow cytometer. Data were analyzed with FACSDIA (BD) and FlowJo software (Tree Star). For general flow cytometry analysis of cell surface markers, cells were cultured as described and stained as above and sorted on a BD Fortessa. Example gating strategy is illustrated in Supplementary Figure 8.

### Mouse FACS analysis and sorting

Mice were euthanized with avertin overdose and lungs were dissected and examined grossly for tumor formation. Tumors were dissected from the lungs of primary mice and tumor tissue was prepared as described^2^. Briefly, tumors were isolated, minced, digested rotating for 1 h at 37 °C with 2 mg/ml collagenase/dispase (Roche), and then filtered twice (100 μm, and then 40 μm) after a 5 min incubation with 0.025 mg/ml DNase. Cell lines were trypsinized and then filtered (40 μm). Single-cell suspensions were stained using anti-CD31-APC (BD Pharmingen 551262), anti-CD45-APC (BD Pharmingen 559864), anti-Epcam-PE-Cy7 (BioLegend 118216), anti-CD24-FITC (BD Pharmingen 553261), anti-Sca-1-APC-Cy7 (BD Pharmingen 560654), with DAPI (Sigma) staining to visualize dead cells. All antibodies were incubated for 15–20 min at 1:100 dilutions. Cell sorting was performed with a Beckman Coulter/Cytomation MoFlo or a BD FACS Aria, and data were analyzed with the FlowJo software (Tree Star Inc.). Example gating strategy is illustrated in Supplementary Figure 9.

### Quantitative RT-PCR

qPCR was carried out as previously described^2^. Cultured cells or tissues were harvested and dissociated according to their specific protocols. RNA was isolated using the Absolutely RNA Microprep Kit (Agilent). Complementary DNA was made using the SuperScript III Kit (Invitrogen) and analyzed using TaqMan Assays (Applied Biosystems) with a StepOnePlus™ Real-Time PCR System (Applied Biosystems) and software as per the manufacturer’s recommendations. *Gapdh* (4352339E) was used as an endogenous control for normalization.

### Organoid-forming assays

For organoids derived from cultured adenocarcinoma cells or primary tumors, dissociated single cells were re-suspended in a 1:1 ratio of GFR Matrigel (Corning) and DMEM/F12 supplemented with 1× ITS (Corning) and 95 µl of cell mixture was plated in 6.5 mm, 0.4 µM membrane transwell plates (CoStar). Either 1000 (cell lines) or 5000 cells (Primary cells) were plated per well. Four hundred and ten miroliters of DMEM/F12 was plated beneath the transwell and was refreshed every 48 h. At the end of the culture period, organoids were counted.

### Tumor transplant assays

Intratracheal transplants of primary sorted tumor cells were performed as described^1,2^. Mice were monitored for signs of lung tumor onset and euthanized for gross and histological analysis upon signs of distress. Tail vein injections were performed as previously described^2^, cells were re-suspended in PBS, and mice were sacrificed 2 weeks after injection for analysis of lung metastasis. For limiting dilution assays, cells were re-suspended in PBS and diluted to the appropriate concentrations. Cell suspensions were then mixed 1:1 to Matrigel and injected subcutaneously into immunocompromised mice as previously described^2^. Mice were sacrificed after 3 weeks and tumors were measured and analyzed.

### Western blotting

Western blotting was performed as was previously described^38^, using the following antibodies and concentrations: H3K9me2 (Ab1220, Abcam, 1:1000) and histone H3 (ab1791, Abcam, 1:10,000). Uncropped scans are displayed in Supplementary Figure 10.

### Proliferation assays

Cells were plated at 500 cells per well in 24-well plates and grown for 192 h, with media changes every 48 h and wells fixed at timepoints with 10% formalin. Fixed cells were stained with Crystal violet and eluted stain was measured for OD 600 absorbance on a Synergy Neo plate reader.

### Microarray analysis

Microarray analysis was performed as previously described^2^.

### ChIP and ChIP-seq

Treated cell lines were crosslinked with 1% formaldehyde in PBS for 10 min at room temperature (RT), washed in 5 mg ml^−1^ bovine serum albumin in PBS and then in just cold PBS, re-suspended in lysis buffer (50 mM Tris-HCl, pH 8.1, 10 mM EDTA, 1% sodium dodecyl sulfate (SDS), 1× complete protease inhibitors (Roche)) and sonicated with the Covaris M220 sonicator to obtain chromatin fragment lengths of 200–500 bp judged by Bioanalyzer DNA High Sensitivity Kit (Agilent). Fragmented chromatin was diluted in immunoprecipitation (IP) buffer (20 mM Tris-HCl, pH 8.1, 150 mM NaCl, 2 mM EDTA, 1% Triton X-100) and incubated overnight at 4 °C with Protein G magnetic beads (Dynabeads: Life Technologies) that had been pre-incubated with H3K9me2 (Abcam, ab1220, 62.5 ng/µl) or H3K9me1 (Abcam, ab8896, 62.5 ng/µl) antibodies. Immunoprecipitates were washed six times with wash buffer (50 mM HEPES, pH 7.6, 0.5 M LiCl, 1 mM EDTA, 0.7% Na deoxycholate, 1% NP-40) and twice with TE buffer. Immunoprecipitated (or no IP input) DNA was treated with RNase A and proteinase K on the beads, recovered in 1% SDS and 0.1 M NaHCO_3_ over a period of 5 h at 65 °C, and column purified with DNA clean and concentrator-25 (Zymo Research). One to ten nanograms of DNA were used for the library construction using NEBNext Ultra DNA Library Prep Kit (NEB E7370). Sequencing was performed on a NextSeq (Illumina) for 38 nucleotides from paired ends according to the manufacturer’s instructions.

Sequenced reads were mapped to reference mouse genome build 38 (mm10), using bowtie2 aligner. H3K27Ac-modified regions were identified using MACS (version 1.4.2) as previously described^43^, with a *P* value cutoff of 1E−5 and with default values for other parameters. Computer code is available upon request. Quantitative differences of histone modification between treated and non-treated cell lines were analyzed using the MAnorm algorithm with a *P* value cutoff of 1E−10 and >1 log 2 fold change.

Wiggle files with a 10 bp resolution for H3K9me2 and H3K27ac modifications were generated by MACS v1.4.2 with tag shift and then rescaled to a larger of total number of uniquely alignable sequences in two data sets. Histone modification profiles were then generated with the “heatmap” function or the SitePro module in Cistrome analysis pipeline (http://cistrome.org/). Wiggle files were also visualized in the Integrative Genomics Viewer^44^. ChIP-seq experiments were performed with an *n* of four biological replicates

For ChIP-PCR, purified DNA from IPs as described using H3K9me2 (Ab1220, Abcam), histone H3 (ab1791, Abcam), or IgG antibodies and input DNA was amplified using the SYBR Green (Applied Biosystems) with a StepOnePlus™ Real-Time PCR System (Applied Biosystems) and software as per the manufacturer’s recommendations. The primer sequences used were as follows: *Gapdh* TSS—FWD, 5′-CTC TCT TTG GAC CCG CCT CAT TT-3′ and REV, 5′-GAG TCC TAT CCT GGG AAC CAT CAC-3′; *Sca-1* TSS—FWD, 5′-TAA CT TTT CCA GGC CAT CAT-3′ and REV, 5′-GTT CCT CCC CAA CTG CTA TAA-3′; *Mmp10* TSS—FWD, 5′-TGA CCT AAA CAC CGA CTC CCT-3′ and REV, 5′-AGG ATG GCT AGT GGC TCC AT-3′.

### RNA-sequencing

RNA-seq was performed by the Molecular Biology Core Facilities at the Dana-Farber Cancer Institute. RNA libraries were prepared using Illumina TruSeq Stranded mRNA Sample Preparation Kits from 500 ng of purified total RNA according to the manufacturer’s protocol. The resultant dsDNA libraries were quantified by Qubit fluorometer, Agilent TapeStation 2200, and quantitative reverse transcription PCR using the Kapa Biosystems Library Quantification Kit according to the manufacturer’s protocols. Uniquely indexed libraries were pooled in equimolar ratios and sequenced on a single Illumina NextSeq500 run with single-end 75 bp reads by the Dana-Farber Cancer Institute Molecular Biology Core Facilities.

Sequenced reads were aligned to the UCSC mm9 reference genome assembly and gene counts were quantified using STAR (v2.5.1b)^45^. Differential gene expression testing was performed by DESeq2 (v1.10.1)^46^ and normalized read counts (RPKM) were calculated using cufflinks (v2.2.1)^47^. RNA-seq analysis was performed using the VIPER snakemake pipeline (https://bitbucket.org/cfce/viper/).

### Immunofluorescence and immunohistochemistry

Immunofluorescence of tumor sections was performed as previously described^2^, with antibodies for pro-SPC (1:200, Santa Cruz), G9a (ab185050, Abcam, 1:200), Notch3 (5276S, Cell Signaling, 1:100), Mmp10 (ab59437, Abcam, 1:100), Mmp13, (ab39012, Abcam, 1:100), and Mcpt4 (ab92368, Abcam, 1:100). Immunohistochemistry was performed as described^2^ with anti-Ki-67 (1:10,000, Novocastra) antibodies and developed using Vectastain Elite ABC Kit (Vector Labs). Imaging was performed with a Nikon 90i camera and NIS-Elements software and processed with NIS-Elements and Adobe Photoshop.

### Mice and tissues

*Lox-Stop-Lox-Kras*^*G12D*^;*p53*^*fl/fl*^ and *Lox-Stop-Lox-Kras*^*G12D*^ mice^48,49^ were maintained in virus-free conditions in the 129 SvJae background. All mouse experiments were approved by the BCH Animal Care and Use Committee and by the Dana-Farber Cancer Institute Institutional Animal Care and Use Committee, both accredited by AAALAC, and were performed in accordance with relevant institutional and national guidelines and regulations. Lung tissue preparation was as described^2^ and sections were analyzed for tumors by at least two investigators including a pathologist with expertise in murine lung cancer. Tumor burden, the percentage of lung filled with tumor, was calculated using ImageJ to measure total lung and tumor area from hematoxylin and eosin (H&E)-stained paraffin sections from each mouse. For metastases, heart, liver, spleen, kidneys, lymph nodes, and chest wall were also analyzed.

### Kaplan–Meier analysis

Raw gene expression data from the Director’s Challenge human lung adenocarcinoma samples^50^ were obtained (https://caintegrator.nci.nih.gov/caintegrator/). Probe intensities from the Affymetrix U133A platform used in these studies were normalized and modeled using the dChip software^51^ (http://biosun1.harvard.edu/complab/dchip). Kaplan–Meier survival analyses were implemented after the samples were hierarchically clustered based on *EHMT2* (G9a), *KMD3A*, *EHMT1* (Glp), or *PHF8* expression. Survival differences between the two risk groups were assessed using the Mantel–Cox log-rank test.

### Cell viability assays

Cell lines were dissociated, counted, and plated at 3000 cells per well in 96-well plates with drug concentrations of the following doses: JIB-04—0, 1, 3, 10, 30, 100, 300, and 1000 nM; Pemetrexed—0, 1, 10, 100, 300, 1000, 3000, and 10,000 nM. Edge wells were filled with PBS. Fresh media and drug were added after 48 h. After 96 h, cells were fixed with 10% formalin. Fixed cells were stained with Crystal violet and eluted stain was measured for OD 600 absorbance on a Synergy Neo plate reader. Results from independent biological replicate experiments were input into the GraphPad Prism software to extrapolate IC_50_ and s.e.m. of IC_50_ for a given cell line using the nonlinear regression analysis of log(inhibitor) vs. normalized response with a variable slope.

### Invasion and migration assays

Invasion and migration assays were performed with 24-well transwell plates according to the manufacturer’s instructions (Corning). Briefly, cells were serum-starved overnight, harvested with Accutase (Millipore), and counted. The bottom chambers of transwells were filled with 700 μl serum-free media or serum, and 30,000 cells per well were plated in triplicate in 100 μl serum-free media in the top chambers. After a 24-h incubation, nonmigratory cells were removed with a cotton swab, and the migratory cells were fixed with formalin and stained with DAPI. The number of DAPI+ cells were counted and the average number of cells invading or migrating was calculated.

### Mmp activity assay

The Mmp activity assay (ab112126, Abcam) was used as per the manufacturer’s instructions. Briefly, a 70% confluent 10 cm plate of cells was washed with PBS and scraped off in 1 ml 0.5% Triton X in PBS. Cell lysate was incubated in ice for 15 min with occasional inversions, then centrifuged at 10,000 rpm at 4 °C for 10 min. The supernatant was separated and used in the protocol as instructed. Mmps were activated for 40 min at 37 °C and fluorescence was read on a Synergy Neo plate reader.

### Treatment and MRI of orthotopic allograft mouse models

Allograft of lung adenocarcinoma cells was performed as previously described. Tumor bearing mice were randomized into cohorts treated either with vehicle (Kolliphor-EL) or 55 mg/kg JIB-04 3× per week for 7 weeks. The mice were imaged by magnetic resonance imaging biweekly to determine the tumor volume during the respective treatments as described previously in a non-blinded fashion^52^. The tumor burden volume and quantification were reconstructed on the 3D slicer software (http://www.slicer.org). Imaging was performed with a Nikon 90i camera and NIS-Elements software and processed with NIS-Elements and Adobe Photoshop.

### Statistics

The tests used to determine statistical significance are quoted in the appropriate figure legend. *P* values are quoted either within the text or when indicated in the figure itself, and *P* values <0.05 were considered significant. When comparing sets of continuous data from different animals, the Kolmogorov–Smirnov test for normalcy was used to determine if a parametric or non-parametric test was required, except where indicated, all tests were two tailed.

## Electronic supplementary material

Supplementary Information

## Data Availability

Data are available upon reasonable request. The ChIP-seq data and RNA-seq that support the findings of this study have been deposited in NCBI’s Gene Expression Omnibus and are accessible through GEO Series accession numbers GSE100455 and GSE120073. Correspondence and requests for materials should be addressed to C.F.K.
