# Supplementary material for: H3K9 methyltransferases and demethylases control lung tumor-propagating cells and lung cancer progression
Source: Nat Commun. 2018 Nov 19;9:4559. doi: 10.1038/s41467-018-07077-1 (PMC6242814; doi:10.1038/s41467-018-07077-1)
Supplement: Supplementary file 1 — Supplementary Information [file 41467_2018_7077_MOESM1_ESM.pdf]

H3K9 methyltransferases and demethylases control lung tumor-propagating cells and lung cancer progression

Rowbotham et al.

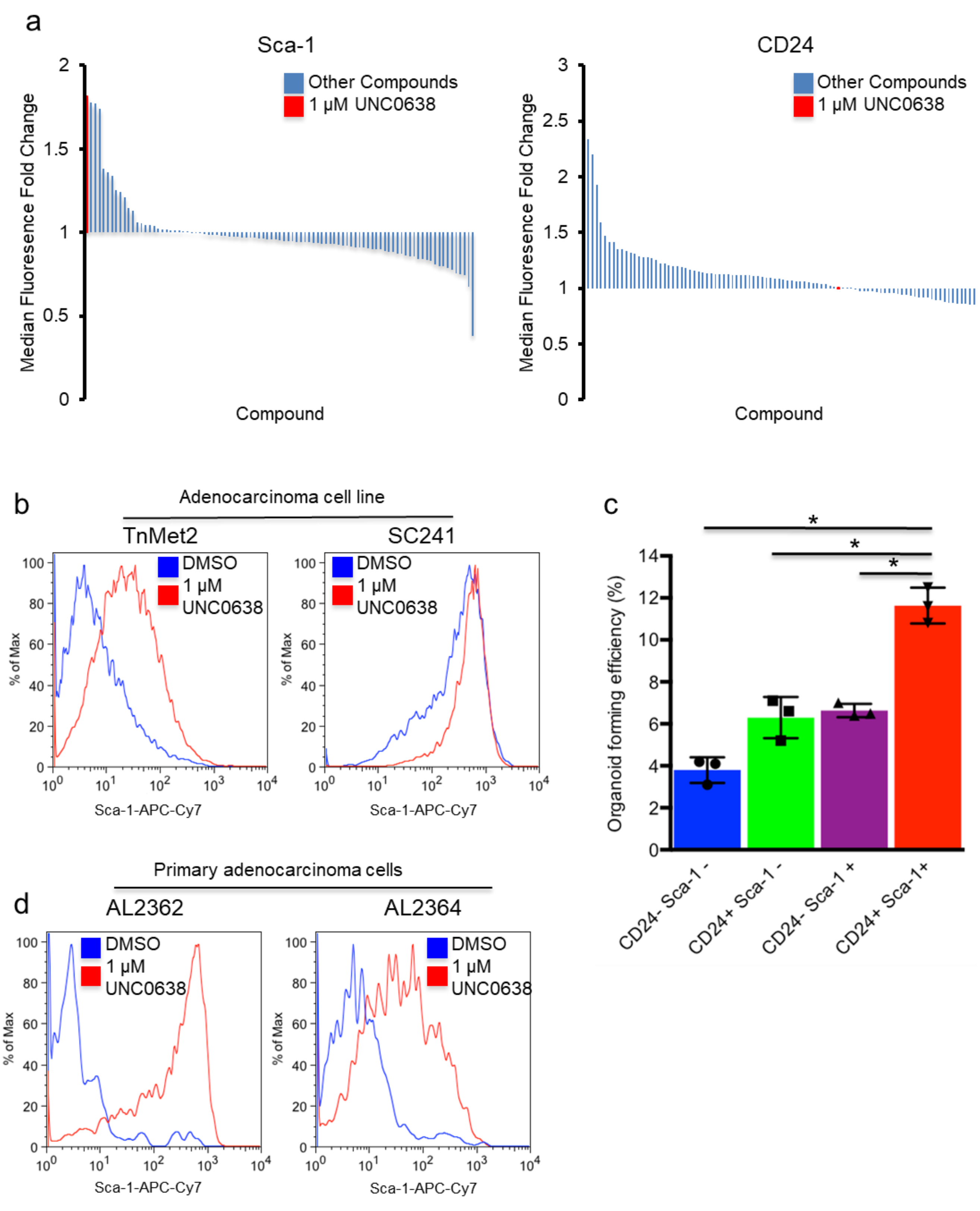

Supplementary Figure 1 a. Bar charts of fold change of either CD24 or Sca-1 fluorescence normalized to DMSO control of drug screened CK1750 cells labeled with fluorescent anti-CD24 and anti-Sca-1 antibodies. Each bar represents an individual compound. **b.** Representative (n=3) FACS histograms of Sca-1 fluorescence of either Sca-1 low (TnMet2) or Sca-1 high (SC241) adenocarcinoma cells following 96 hrs treatment with 1 μM UNC0638 or vehicle control, sorted for live single cells. **c.** Bar chart of % of cells forming organoids 10 days after adenocarcinoma cells were FACS sorted (gated for single, live cells) for CD24 and Sca-1 and 2000 cells were plated in 3D culture. Error bars denote standard deviation, *=P<0.05, T test, n=3. **d.** FACS histograms of Sca-1 fluorescence of dissociated organoids from primary adenocarcinoma tumors following 10 days growth with either 1 μM UNC0638 or DMSO, gated for single live cells.

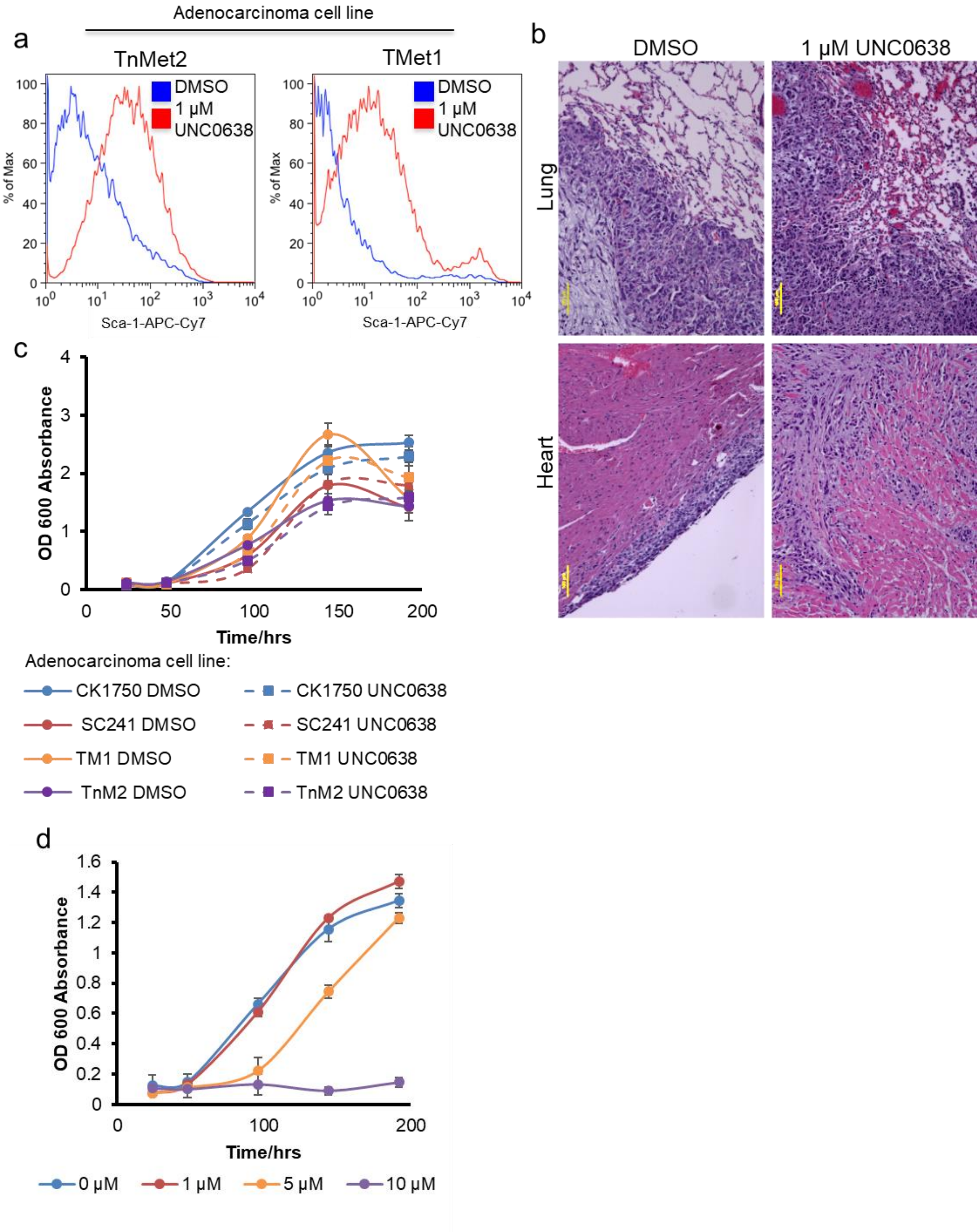

Supplementary Figure 2 a. Representative (n=3) FACS histograms of Sca-1 fluorescence of adenocarcinoma cells following 96 hrs treatment with 1 μM UNC0638 or vehicle control, sorted for live single cells, immediately prior to intravenous injection. **b.** Representative images of tumors in H&E stained sections of indicated tissues from recipients of intravenously injected adenocarcinoma cells grown with DMSO or 1 μM UNC0638. Scale bar = 100 μm. **c.** OD 600 absorbance of crystal violet stained cultures of adenocarcinoma cells grown continuously with either 1 μM UNC0638 or DMSO fixed at the indicated timepoints. Error bars denote standard deviation, n=3. **d.** OD 600 absorbance of crystal violet stained cultures of CK1750 adenocarcinoma cells grown continuously with indicated concentrations of UNC0638 and fixed at the indicated timepoints. Error bars denote standard deviation n=3.

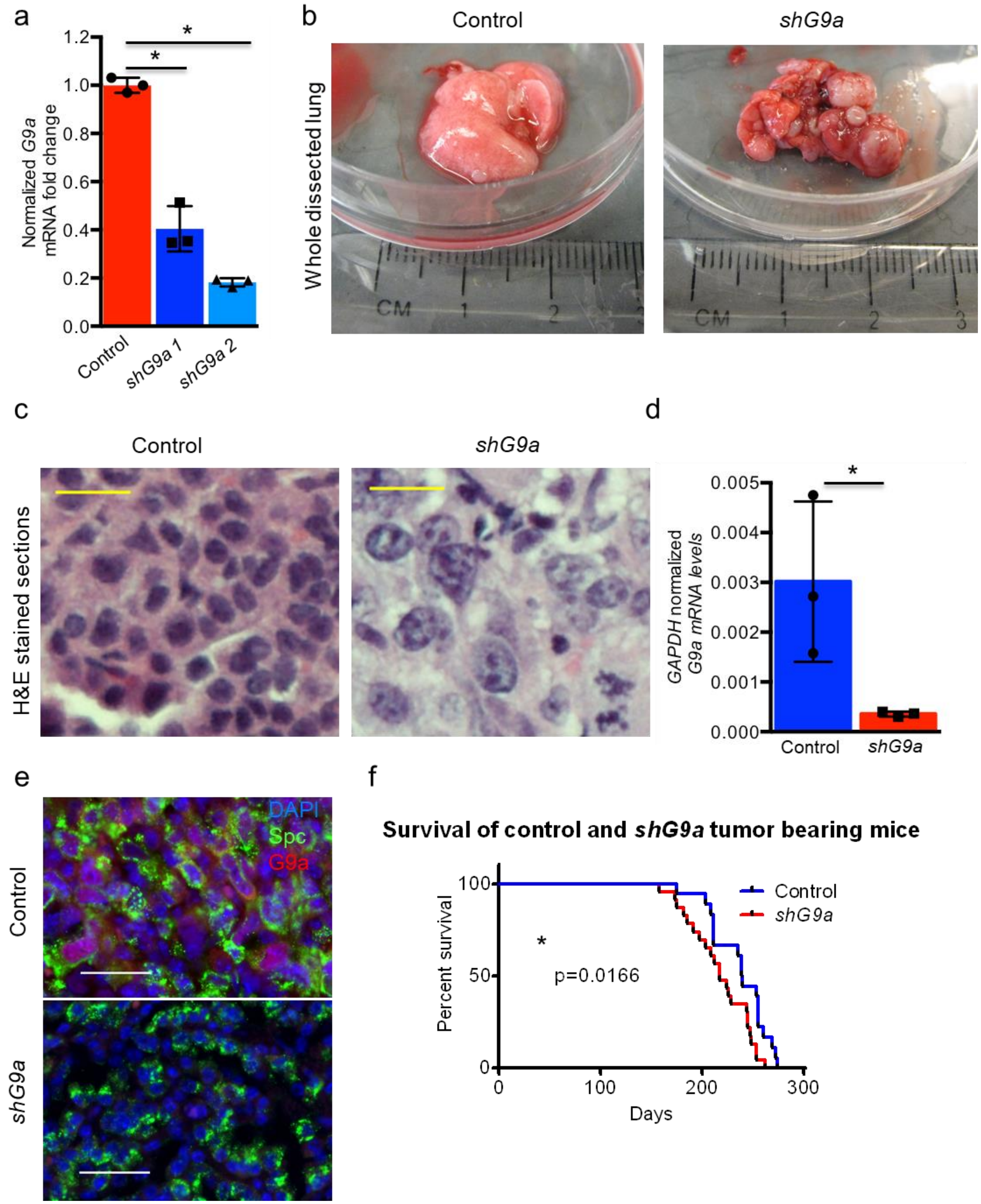

Supplementary Figure 3 a. Representative bar chart of fold change of *Gapdh*-normalized G9a mRNA levels in 3T3 cells transfected with shG9a lentiviral vectors compared to cells transfected with control lentiviral vector. Error bars denote standard deviation, *=P<0.05, Fisher's T test, n=3 technical replicates. Mann-Whitney test, n=5. **b.** Representative images of whole lungs harvested from recipients of Control and *shG9a* lentiviruses. **c.** Representative H&E stained sections of control and *shG9a* lung tumors, n=16 and n=24 mice respectively. Scale bar = 25 µm. **d.** Bar chart of fold change of *Gapdh*-normalized G9a mRNA levels in lung adenocarcinoma cells isolated from control and *shG9a* tumors. Error bars denote standard deviation, *=p<0.05, Fisher's T test, n=3. **e.** Representative Immunofluorescence of sections of lung tumors from recipients of control and *shG9a* lentiviruses, stained with indicated antibodies. Scale bar = 25 µm. **f.** Kaplan-Meier survival curve of *shG9a* and control mice measuring time from tumor initiation to death or experimental sacrifice. *=p<0.05, Mantel-Cox log-rank test.

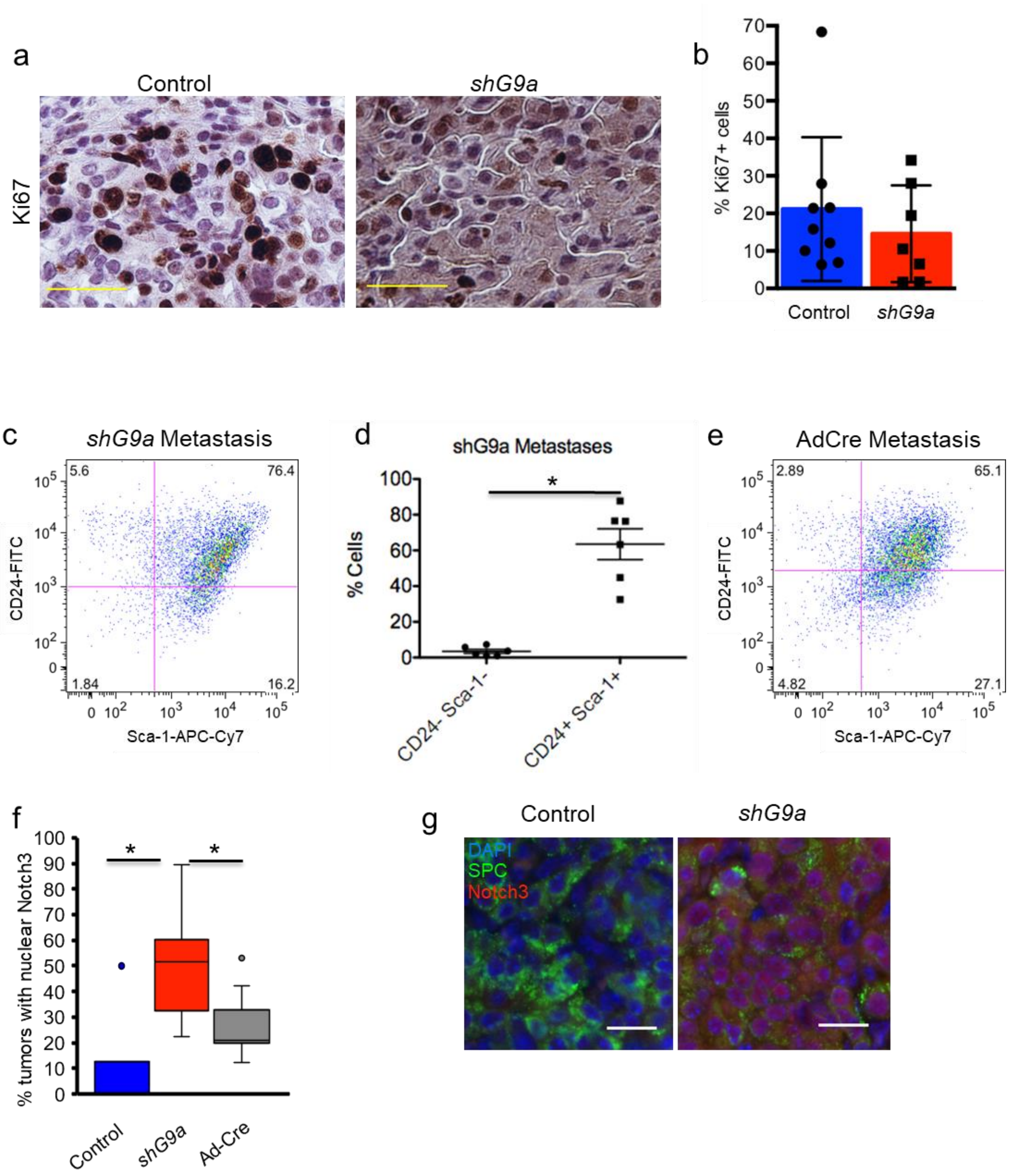

Supplementary Figure 4 a. Representative (n=7) images of Immunohistochemistry of sections of lung tumors from recipients of control and *shG9a* lentiviruses, stained for Ki67. Scale bar = 25 µm. **b.** Bar chart of % of Ki67 stained cells per tumor per mouse in recipients of control and *shG9a* lentiviruses. Error bars denote standard deviation, n=7. **c.** Representative (n=6) FACS plot of a dissociated metastasis from an shG9a lentivirus recipient, gated for live, single, CD31-, CD45-, Epcam+ cells. **d.** Chart showing the median proportion of CD24+ Sca-1+ cells and CD24- Sca-1- cells in metastases from *shG9a* recipients determined by FACS. Each data point represents tumors from an individual mouse. *=$p<0.05$, Mann-Whitney test. **e.** Representative (n=3) FACS plot of a dissociated metastasis from an Adeno-Cre recipient mouse, gated for live, single, CD31-, CD45-, Epcam+ cells. **f.** Box and whisker plot of % of tumors per mouse with Nuclear Notch3 immunostaining in lung sections from recipients of control and *shG9a* lentiviruses. Boxes represent inner quartiles, center line = median, whiskers = 1.5 X IQR. *=$p<0.05$, Mann-Whitney test, n=10. **g.** Representative (n=10) Immunofluorescence of sections of lung tumors from recipients of control and *shG9a* lentiviruses, stained with indicated antibodies. Scale bar = 25 µm.

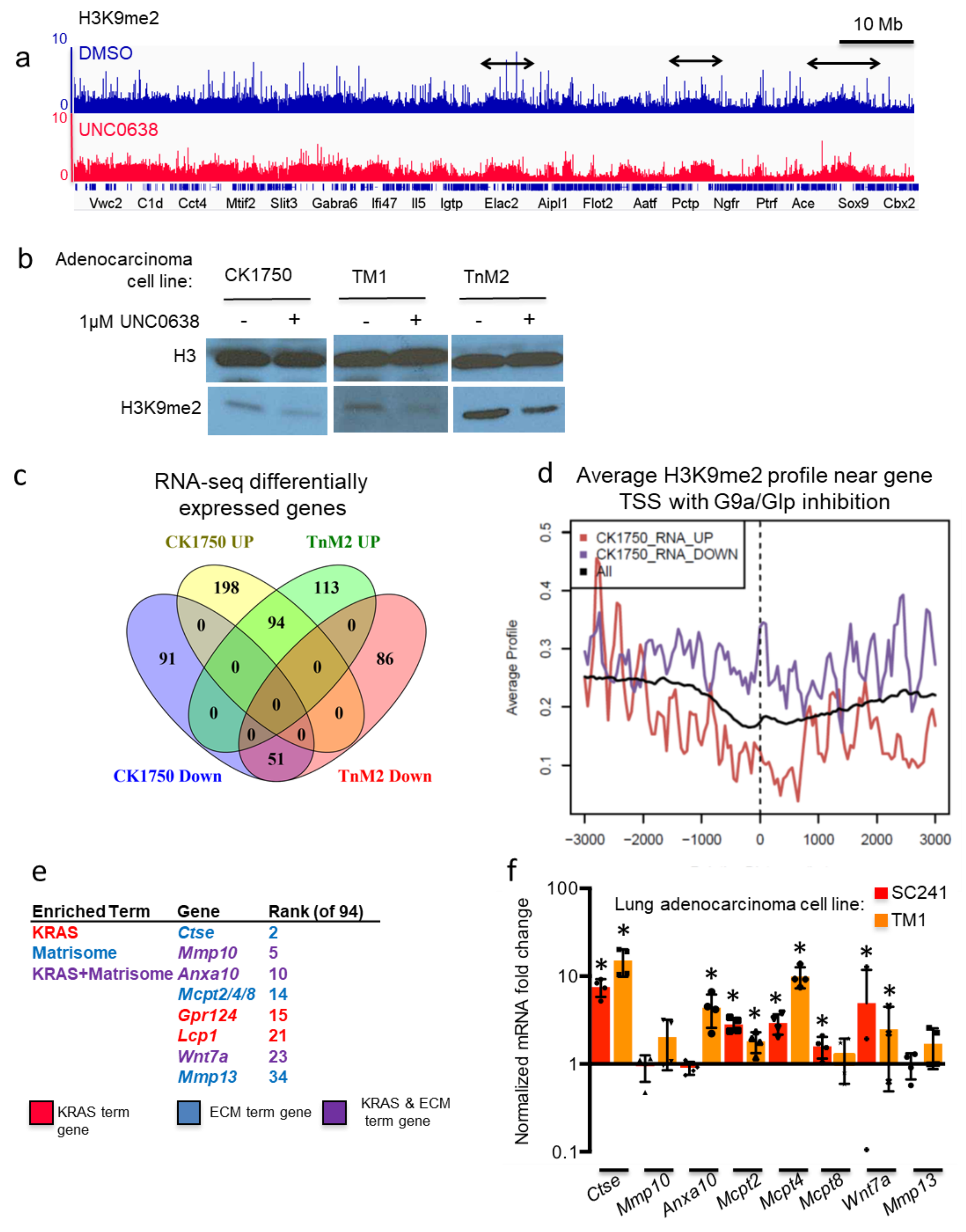

Supplementary Figure 5 a. Representative (n=4) tracks of H3k9me2 ChIP-seq of lung adenocarcinoma cells treated with 1 µM UNC0638 or DMSO. Arrows denote MB sized H3K9me2 domains **b.** Western blot of whole cell lysates from adenocarcinoma cells treated with either 1 µM UNC0638 or DMSO for 96 hrs immunoblotted for the indicated antibodies. **c.** Venn diagram of genes differentially upregulated and downregulated in CK1750 and TnM2 adenocarcinoma cells following 96 hrs treatment with 1 µM UNC0638 or vehicle control. **d.** Average H3K9me2 profiles near the TSS of RNA-seq upregulated and downregulated genes in G9a/Glp inhibited lung adenocarcinoma cells compared to all genes. **e.** List of commonly upregulated genes chosen for further investigation ranked by fold change in gene expression. **f.** Bar chart of fold change of *Gapdh*-normalized mRNA levels of indicated genes in adenocarcinoma cells treated with 1 µM UNC0638 relative to DMSO treated controls. Error bars denote standard deviation, *=p<0.05, Fisher's t test, n=3.

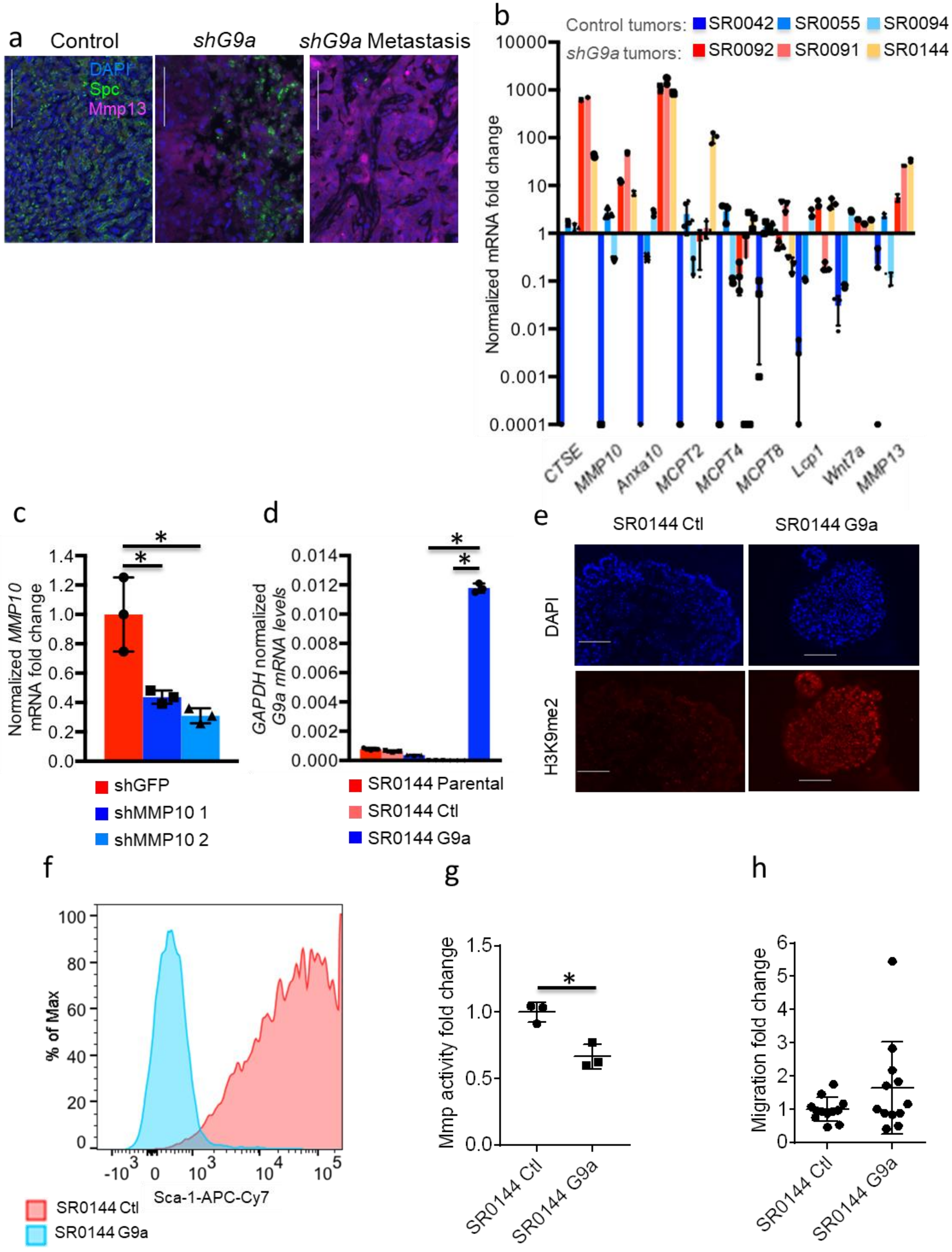
a
Control
shG9a
shG9a Metastasis
DAPI
Spc
Mmp13
b
Control tumors:
SR0042
SR0055
SR0094
shG9a tumors:
SR0092
SR0091
SR0144
Normalized mRNA fold change
10000
1000
100
10
1
0.1
0.01
0.001
0.0001
CTSE
MMP10
Anxa10
MCPT2
MCPT4
MCPT8
Lcp1
Wnt7a
MMP13
c
Normalized MMP10 mRNA fold change
1.4
1.2
1.0
0.8
0.6
0.4
0.2
0.0
*
*
shGFP
shMMP10 1
shMMP10 2
d
GAPDH normalized G9a mRNA levels
0.014
0.012
0.010
0.008
0.006
0.004
0.002
0.000
*
*
SR0144 Parental
SR0144 Ctl
SR0144 G9a
e
SR0144 Ctl
SR0144 G9a
DAPI
H3K9me2
f
100
80
60
40
20
0
% of Max
-10^3
0
10^3
10^4
10^5
Sca-1-APC-Cy7
SR0144 Ctl
SR0144 G9a
g
Mmp activity fold change
1.5
1.0
0.5
0.0
*
SR0144 Ctl
SR0144 G9a
h
Migration fold change
6
5
4
3
2
1
0
SR0144 Ctl
SR0144 G9a

Supplementary Figure 6 a. Representative (n=5) images of *shLuc* control and *shG9a* tumors, immunostained for the indicated for the proteins. Scale bar = 100 µm. **b.** Bar chart of fold change of *Gapdh*-normalized mRNA levels of indicated genes in *shLuc* control and *shG9a* tumors compared to average expression level across all tumors. Error bars denote standard deviation, n=3 technical replicates. **c.** Representative Bar chart of mRNA levels of *Mmp10* normalized to *Gapdh* from *shGFP*, and *shMmp10* SR0144 *shG9a* lung adenocarcinoma cells. Error bars denote standard deviation, *=$p<0.05$, Fisher's T test, n=3 technical replicates. **d.** Representative bar chart of *Gapdh* normalized mouse and human *G9a* mRNA levels in G9a overexpressing, Cre overexpressing control and untransformed SR0144 *shG9a* lung adenocarcinoma cells. Error bars denote standard deviation, *=$p<0.05$, Fisher's T test, n=3 technical replicates. **e.** Representative (n=3) images of control and G9a overexpressing SR0144 *shG9a* lung adenocarcinoma cells, immunostained for H3K9me2 and DAPI. Scale bar = 100 µm. **f.** Representative (n=3) FACS histogram of Sca-1 fluorescence levels of Cre overexpressing and G9a overexpressing SR0144 *shG9a* lung adenocarcinoma cells, gated on live single cells. **g.** Chart of fold change in Mmp enzymatic activity of G9a overexpressing relative to control SR0144 *shG9a* cells measured by an in vitro Mmp activity assay. *=$p<0.05$, Fisher's T test. **h.** Chart of fold change in migration through a migration transwell of G9a overexpressing relative to control SR0144 *shG9a* cells. *=$p<0.05$, Fisher's T test.

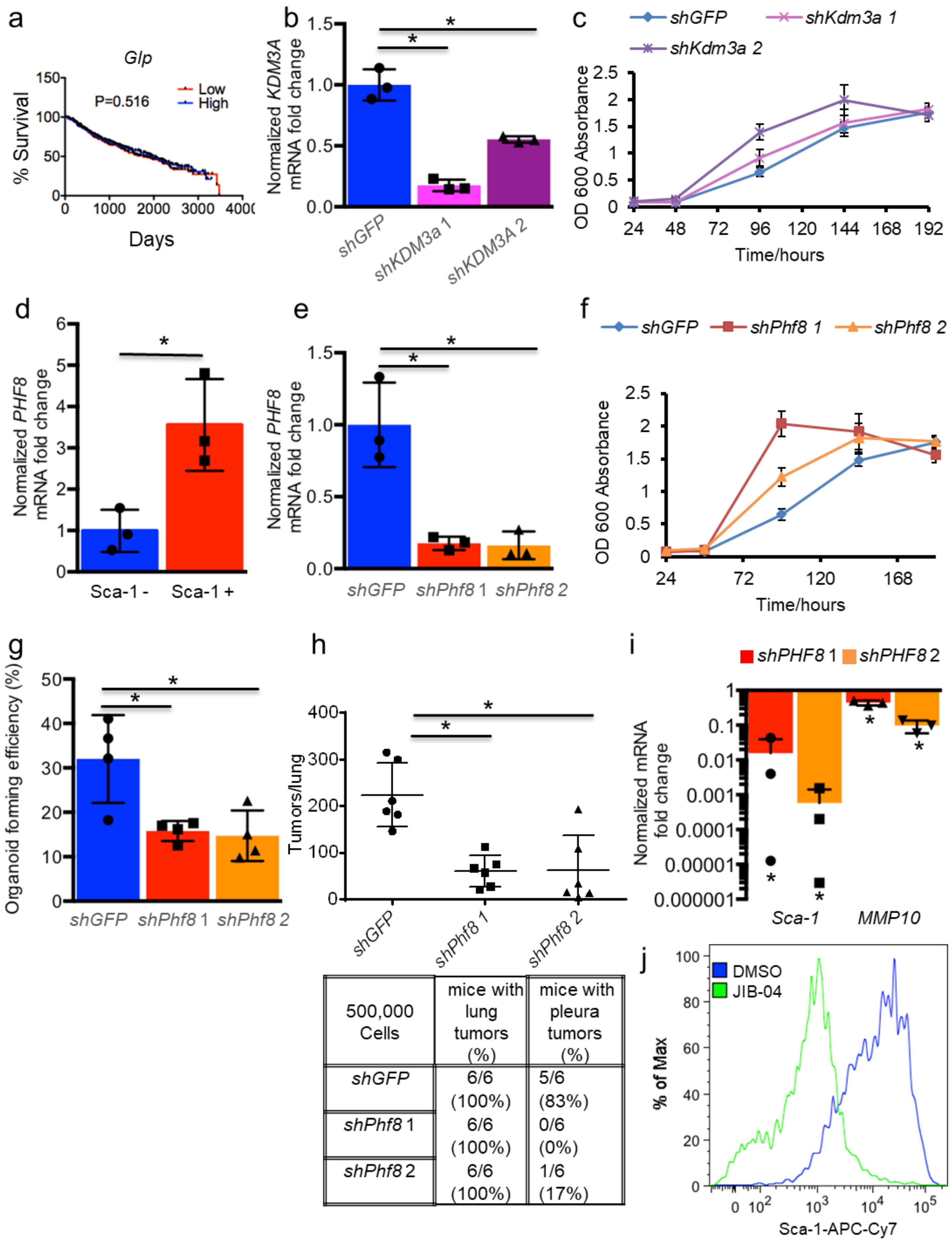

500,000 Cells	mice with lung tumors (%)	mice with pleura tumors (%)
shGFP	6/6 (100%)	5/6 (83%)
shPhf8 1	6/6 (100%)	0/6 (0%)
shPhf8 2	6/6 (100%)	1/6 (17%)

Supplementary Figure 7 a. Kaplan Meier curves of 10 year survival of patients from the director's challenge cohort of early stage lung adenocarcinoma, sorted for high and low expression of *Glp*. p>0.05, Mantel-Cox log-rank test. **b.** Representative qPCR of *Kdm3a* mRNA normalized to *Gapdh* from CK1750 adenocarcinoma cells infected with *shGFP* and *shKdm3a* lentiviruses. Error bars denote standard deviation, *=p<0.05, n=3 technical replicates. **c.** OD 600 absorbance of crystal violet stained cultures of *shGFP* and *shKdm3a* adenocarcinoma cells fixed at the indicated timepoints. Error bars denote standard deviation. **d.** Bar chart of mRNA abundance of *Phf8* normalized to *β-Actin* in Sca-1+ cells (TPCs) relative to Sca-1- cells (non-TPCs) from FACS sorted primary adenocarcinomas, gated for single, live, CD31-, CD45- cells. Error bars denote standard deviation, *=p<0.05, Fisher's T test, n=3. **e.** Representative qPCR of *Phf8* mRNA normalized to *Gapdh* from CK1750 adenocarcinoma cells infected with *shGFP*, *shPhf8* lentiviruses. Error bars denote standard deviation, *=p<0.05, n=3 technical replicates. **f.** OD 600 absorbance of crystal violet stained cultures of *shGFP* and *shPhf8* adenocarcinoma cells fixed at the indicated timepoints. Error bars denote standard deviation. **g.** Bar chart of % of cells forming organoids 10 days after 1000 *shGFP* and *shPHF8* adenocarcinoma cells were plated in 3D culture. Error bars denote standard deviation, *=P<0.05, Fisher's T test, n=4. **h.** Chart of average number of tumors per H&E stained section from recipients of intravenously transplanted *shGFP* and *shPhf8* adenocarcinoma cells. *=p<0.05, Mann-Whitney test, and table of mice with secondary tumors, *=p<0.05, Fisher's exact test. **i.** Bar chart of fold change of *Gapdh*-normalized mRNA levels of indicated genes in *shPhf8* adenocarcinoma cells over *shGFP* control cells. Error bars denote standard deviation, *=p<0.05, Fisher's T test, n=3. **j.** Representative (n=3) FACS histogram of Sca-1 fluorescence of CK1750 adenocarcinoma cells treated with either 100 nM JIB-04 or DMSO for 96 hrs, gated for single, live cells.

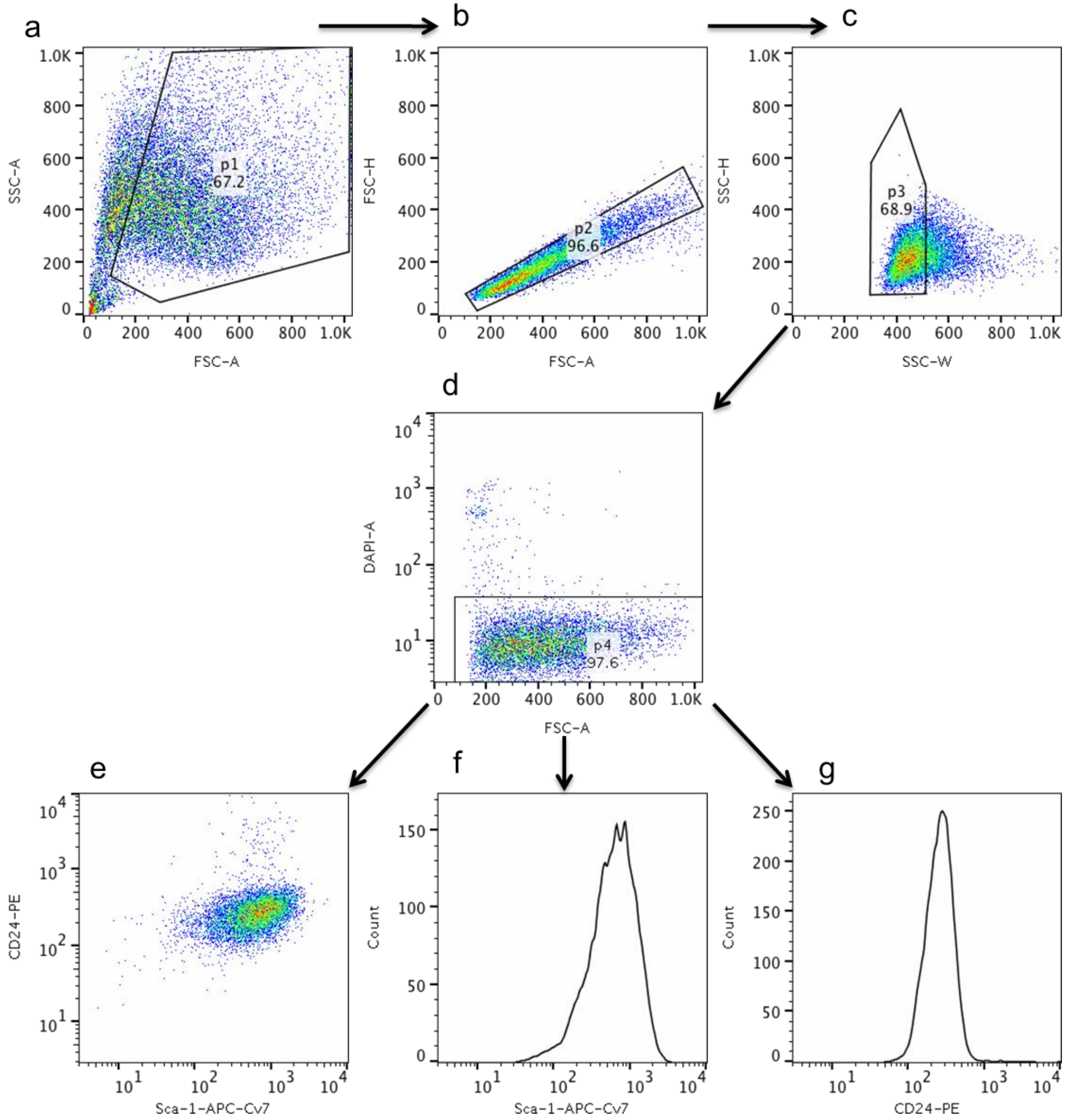

Supplementary Figure 8 a-g. Representative sequential gating strategies for Flow cytometry analysis of in vitro cultured cells. Figure 1 a, Supplementary Figures 1a graphs data from gate represented in panel **e.** Figures 1b, and Supplementary Figures 1b,d, 2a, 6f, 7j plot data represented in panel **f**.

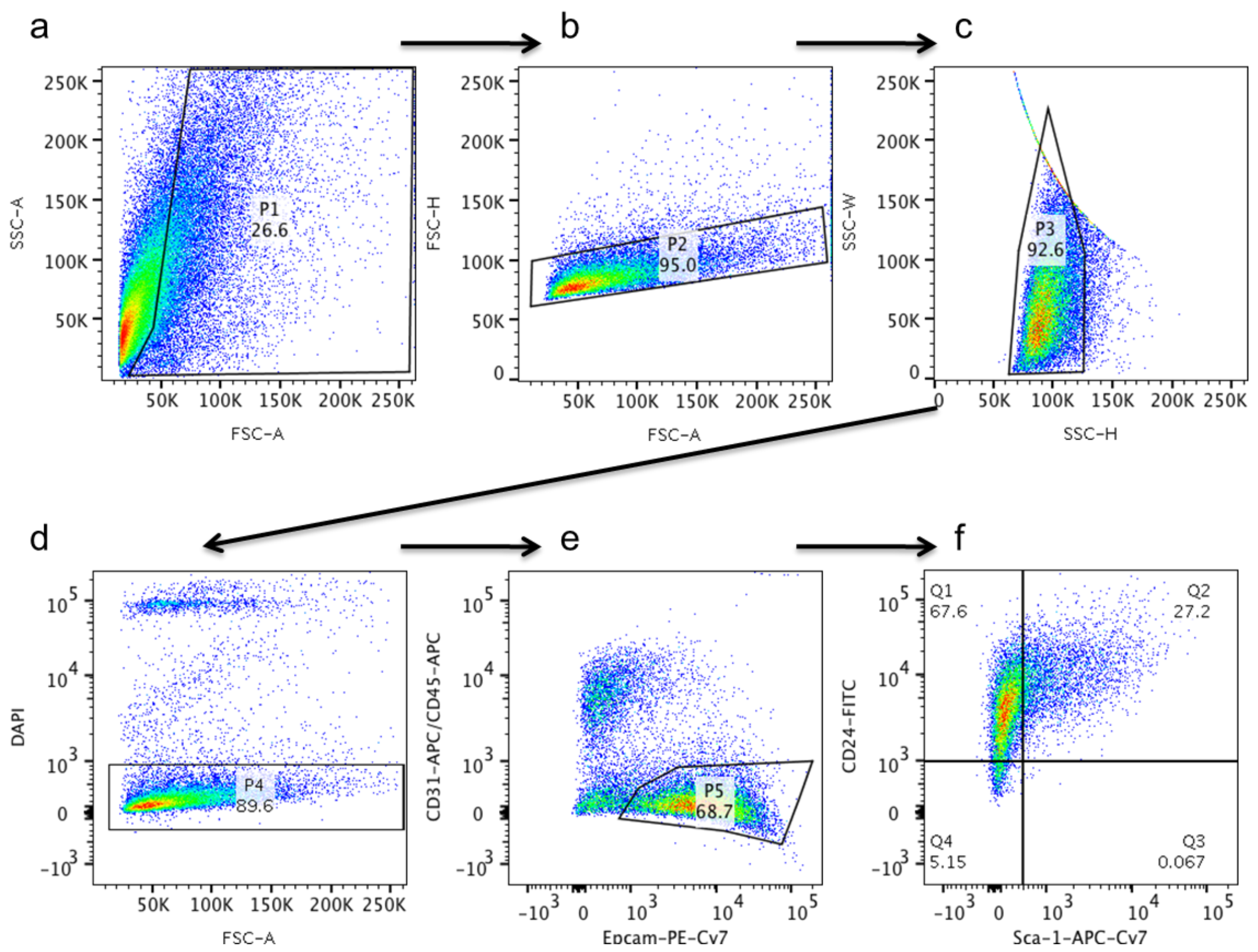

Supplementary Figure 9 a-f. Representative sequential gating strategies for Flow cytometry analysis of mouse tumors. Figures 2e, Supplementary Figure 4c,e plot data represented in panel **f**. Figure 2f, graph data from panel **f** Q2.

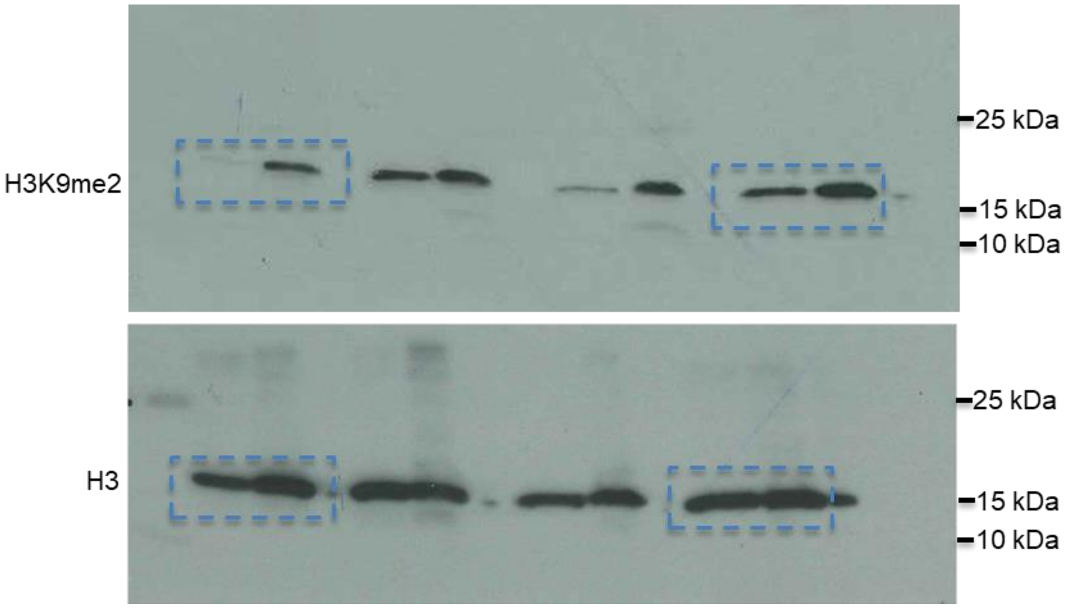

Supplementary Figure 10. Western blots represented in Figure 4g with crops indicated.
